# Age-related unstable transient states and imbalanced activation proportion of brain networks in people with autism spectrum disorder: A resting-state fMRI study using coactivation pattern analyses

**DOI:** 10.1162/netn_a_00396

**Published:** 2024-12-10

**Authors:** Yunge Zhang, Lin Lin, Dongyue Zhou, Yang Song, Abigail Stein, Shuqin Zhou, Huashuai Xu, Wei Zhao, Fengyu Cong, Jin Sun, Huanjie Li, Fei Du

**Affiliations:** Central Hospital of Dalian University of Technology, Dalian, China; School of Biomedical Engineering, Faculty of Medicine, Dalian University of Technology, Dalian, China; Department of Cognitive Neuroscience, Donders Institute for Brain, Cognition, and Behaviour, Radboud University Medical Centre, Nijmegen, The Netherlands; McLean Imaging Center, McLean Hospital, Harvard Medical School, Belmont, MA, USA; School of Software, Henan Polytechnic University, Jiaozuo, China; Faculty of Information Technology, University of Jyväskylä, Jyväskylä, Finland; Key Laboratory of Social Computing and Cognitive Intelligence (Dalian University of Technology), Ministry of Education, Dalian, China; Dalian Woman and Children’s Medical Group, Dalian, China; Psychotic Disorders Division, McLean Hospital, Harvard Medical School, Belmont, MA, USA

**Keywords:** Autism spectrum disorder, Triple network model, Default model network, Coactivation pattern, Resting-state functional MRI

## Abstract

The atypical static brain functions related to the executive control network (ECN), default mode network (DMN), and salience network (SN) in people with autism spectrum disorder (ASD) has been widely reported. However, their transient functions in ASD are not clear. We aim to identify transient network states (TNSs) using coactivation pattern (CAP) analysis to characterize the age-related atypical transient functions in ASD. CAP analysis was performed on a resting-state fMRI dataset (78 ASD and 78 healthy control (CON) juveniles, 54 ASD and 54 CON adults). Six TNSs were divided into the DMN-TNSs, ECN-TNSs, and SN-TNSs. The DMN-TNSs were major states with the highest stability and proportion, and the ECN-TNSs and SN-TNSs were minor states. Age-related abnormalities on spatial stability and TNS proportion were found in ASD. The spatial stability of DMN-TNSs was found increasing with age in CON, but was not found in ASD. A lower proportion of DMN-TNSs was found in ASD compared with CON of the same age, and ASD juveniles had a higher proportion of SN-TNSs while ASD adults had a higher proportion of ECN-TNSs. The abnormalities on spatial stability and TNS proportion were related to social deficits. Our results provided new evidence for atypical transient brain functions in people with ASD.

## Author Summary

This article reveals the age-related atypical transient brain functions in autistic people. The default mode network dominates major states during rest, while the executive control network and salience network dominates minor states. Major states have more a stable coactivation patterns and higher proportion than minor states. The spatial stability would increase with age in healthy controls but not in autistic people. Meanwhile, autistic people spend less time on major states but more time on minor states. In addition, the unstable transient states and imbalanced activation proportion of brain networks are correlated with the social deficits. Although this work is limited by that single scanning data of only involving male participants with normal intelligence, it provides new transient aspect for atypical brain function in autistic people.

## INTRODUCTION

Autism spectrum disorder (ASD) is a neurodevelopmental disorder with two core symptom domains—impaired social communication deficit and restrictive interests/repetitive behaviors (Sharma, Gonda, & Tarazi, 2018). Currently, the biological mechanism of ASD is still unclear, and there is no specific biomarker to help with early diagnosis. Functional magnetic resonance imaging (fMRI) has become an important and potential tool for studying the mechanism of atypical brain function in ASD. In particular, resting-state fMRI scanning is used wildly since it is easier for autistic people to participate. The triple network model (Liang, Zou, He, & Yang, 2016; Menon, 2011; Uddin, 2015), which is composed of the default mode network (DMN), executive control network (ECN), and salience network (SN), has been applied to detect impaired cognitive, perceptual, affective, and social functioning. Since these domains of functioning are highly related to the core symptoms of ASD, many fMRI studies of ASD have focused on this model and reported atypical brain activity.

Static functional connectivity (FC), defined as the correlation coefficient of time courses of BOLD signal between two regions, is the most common feature used to study atypical brain function in ASD (Abbott et al., 2016; Bi, Zhao, Xu, Sun, & Wang, 2018; Hogeveen, Krug, Elliott, & Solomon, 2018). However, static FC is based on the entire time course of fMRI scan and lacks information about network connections that vary over time (Allen et al., 2014; Calhoun, Miller, Pearlson, & Adalı, 2014). It has been proven that dynamic FC (dFC) is more sensitively correlated with different physiologic and pathologic brain states than static FC (Liégeois et al., 2019). The most common and traditional dFC method is to apply a sliding time window to segment the BOLD time course into short frames to capture connection within each time window. Although traditional dFC overcomes some limitations of static FC analysis, there is a concern that the different window sizes used in traditional dFC can have a substantial impact on the results (Vergara, Mayer, Damaraju, & Calhoun, 2017).

Dynamic brain activity can also be captured by coactivation pattern (CAP) analyses more precisely and can embody transient brain activities (Janes, Peechatka, Frederick, & Kaiser, 2020; Liu & Duyn, 2013b; Liu, Zhang, Chang, & Duyn, 2018). CAP analysis uses clustering to reflect common activation patterns with a single-frame temporal resolution, which are defined as transient network states (TNSs) of the brain. This method is not limited by window size and is less affected by noise. The CAP analysis can be implemented based on seeds or whole-brain approach (Liu et al., 2018). The seed-based CAP approach focuses on the coactivation between a chosen seed and other regions, only frames showing strong activation of the seed are extracted to perform the clustering. By this approach, Marshall et al. (2020) reported a lower dwell time (the amount of time spent in a TNS over the time series) of coactivation of DMN and ECN in children with ASD. Notably, the seed-based CAP approach has obvious limitations. First, it is based on prior assumptions, and second, the frame extraction breaks the continuity of time. On the contrary, the whole-brain CAP approach is performed with all frames of all regions of interest. However, the study of ASD that used the whole-brain CAP approach is rare. Using this approach, Kupis et al. (2020) reported a correlation between social deficits and dwell time of an ECN-activating TNS in children with ASD, but only six regions of interest were studied. In addition, previous CAP studies only focused on the temporal features of TNSs, while the spatial stability of TNSs has not been well-studied. Since the TNSs are obtained from clustering, there should be spatial variances among all frames within one TNS, which represent spatial stability of it. Which kind of TNSs are stable and which regions are activated steadily in one TNS are not yet clear. The stability of dFC (named as temporal variability) is reported to predict behavior more accurately than static FC in a typical population (Jia, Hu, & Deshpande, 2014) and shows promise as a potential biomarker in ASD (Chen, Nomi, Uddin, Duan, & Chen, 2017). It is not clear whether the spatial stability of TNSs is also related to social communication deficits in ASD.

In this study, we will use the whole-brain CAP approach with a fine brain parcellation to analyze a well-matched dataset (Di Martino et al., 2017). Further, we will examine the atypical transient brain function of people from both spatial stability and temporal features in juveniles and adults with ASD. The primary aim is to address the three following research questions: (a) Does spatial stability differ across TNSs? (b) Does the spatial stability and temporal features of TNSs differ between people with ASD and healthy controls (CON), and is there any age effect involved? (c) What is the relationship between atypical transient dynamic brain functioning and social deficits in people with ASD?

## MATERIALS AND METHODS

### Subjects

Data from the ABIDE II dataset (Di Martino et al., 2017) was used for this research, including 1,114 subjects from 16 sites. High-resolution, T1-weighted structural images and resting-state fMRI data were collected for each subject. For this CAP analysis, exclusion criteria were the following: (a) female subjects, (b) full IQ under 80, (c) obvious artifacts in T1-weighted data, and (d) fMRI head motion larger than its voxel size. The number of female subjects and subjects with full IQ under 80 is low in each site, so these subjects were excluded. To reduce the effect of covariates, subjects in the ASD and CON groups were matched for age and full IQ at each site; each site also included more than 10 subjects in each group. This selection resulted in 156 juvenile subjects from four sites (6–17.8 years old, 78 ASD and 78 CON from Georgetown University, GU; San Diego State University, SDSU; New York University Langone Medical Center, NYU; Stanford University, SU) and 108 adult subjects from three sites (older than 18 years old, 54 ASD and 54 CON from Indiana University, IU; Olin Neuropsychiatry Research Center, ONRC; Barrow Neurological Institute, BNI). Participant demographics are provided in Table 1. People with ASD always show stronger head motion in fMRI data (Caballero, Mistry, & Torres, 2020). To ensure an adequate sample size, this study did not guarantee the matching of head motion levels within site between two groups; the ASD group had a significantly larger motion than the CON group in NYU. To reduce the effect of head motion, the mean absolute motion was one covariate in statistical analyses.

**Table 1.** The demographic information

	ASD group	CON group	*p* value
**GU (TR = 2,000 ms, 147 frames)**			
Num of subjects	20	20	
Age [years] (8.06–13.88)	11.29 ± 1.42	10.82 ± 1.73	0.5931^a^
Full IQ (96–149)	107.89 ± 13.95	112.07 ± 12.15	0.2373^a^
Mean absolute motion [mm] (0.09–0.81)	0.31 ± 0.22	0.25 ± 0.12	0.3695^a^
Eye status	Closed	Closed	
**SDSU (TR = 2,000 ms, 175 frames)**			
Num of subjects	21	21	
Age [years] (8–17.8)	13.23 ± 3.21	13.43 ± 3.21	0.8374^a^
Full IQ (80–130)	102.90 ± 11.90	104.10 ± 8.98	0.7164^a^
Mean absolute motion [mm] (0.06–0.98)	0.34 ± 0.21	0.30 ± 0.26	0.5882^a^
Eye status	Closed	Closed	
**NYU (TR = 2,000 ms, 175 frames)**			
Num of subjects	24	24	
Age [years] (5.89–14.98)	9.05 ± 2.42	8.99 ± 2.02	0.9289^a^
Full IQ (88–138)	109.83 ± 14.44	115.50 ± 13.73	0.1703^a^
Mean absolute motion [mm] (0.13–0.89)	0.34 ± 0.21	0.23 ± 0.09	0.0314^a^
Eye status	Closed	Closed	
**IU (TR = 813 ms, 428 frames)**			
Num of subjects	13	13	
Age [years] (18–37)	22.69 ± 5.48	25.08 ± 5.53	0.2805^a^
Full IQ (80–135)	116.15 ± 13.67	116.15 ± 10.40	1.0000^a^
Mean absolute motion [mm] (0.09–0.41)	0.18 ± 0.05	0.17 ± 0.09	0.6265^a^
Eye status	Closed	Closed	
**ONRC (TR = 475 ms, 942 frames)**			
Num of subjects	13	13	
Age [years] (18–28)	20.69 ± 3.01	23.15 ± 3.51	0.0668^a^
Full IQ (86–138)	110.46 ± 14.69	114.23 ± 12.21	0.4837^a^
Mean absolute motion [mm] (0.08–0.80)	0.30 ± 0.21	0.28 ± 0.17	0.7622^a^
Eye status	Closed	Closed	
**BNI (TR = 3,000 ms, 115 frames)**			
Num of subjects	28	28	
Age [years] (18–64)	38.11 ± 15.98	40.32 ± 14.83	0.5931^a^
Full IQ (85–141)	107.89 ± 13.95	112.07 ± 12.15	0.2373^a^
Mean absolute motion [mm] (0.06–0.79)	0.21 ± 0.15	0.23 ± 0.16	0.6318^a^
Eye status	Open	Open	
**SU (TR = 2,000 ms, 175 frames)**			
Num of subjects	13	13	
Age [years] (8.43–12.99)	10.61 ± 1.08	10.98 ± 0.99	0.3800^a^
Full IQ (93–137)	115.15 ± 16.71	112.85 ± 13.86	0.7049^a^
Mean absolute motion [mm] (0.06–0.79)	0.29 ± 0.23	0.34 ± 0.22	0.5533^a^
Eye status	Open	Open	

Data are expressed as mean ± standard deviation. Num, number.

^a^ Two-sample *t* test.

### Data Preprocessing

The scanner information and protocols differ between sites. The TR and number of frames are listed in Table 1, and details can be found on the ABIDE II website (https://fcon_1000.projects.nitrc.org/indi/abide/). Data preprocessing was conducted using *FSL* (Smith et al., 2004; Woolrich, Ripley, Brady, & Smith, 2001). For the fMRI data, five steps were conducted with *FSL*: (a) removal of the first five frames, (b) nonlinear registration to a 2-mm MNI template, (c) motion correction, (d) spatial smoothing (6 mm at FWHM), and (e) high-pass temporal filtering (cutoff, 0.01 Hz). The following steps were carried out using *MATLAB*: (f) nuisance regression, including six head motion parameters, mean white matter signal, mean cerebrospinal fluid signal, and global signal regression; (g) detrend, including demean, linear, and quadratic trends; and (h) band-pass temporal filtering (0.01–0.1 Hz).

### Coactivation Patterns

Using the whole-brain approach described in previous studies (Liu et al., 2018; Yang et al., 2021), the CAP analysis was performed using homemade scripts in *MATLAB*. First, a 400-node cortical parcellation (Schaefer et al., 2018) was used to extract time courses. This parcellation is based on the seven-network model proposed by Yeo et al. (2011), which divides the whole cortex into the ECN, DMN, dorsal attention network (DAN), limbic network (Lim), SN, somatomotor network (SM), and visual network (Vis). Next, two-dimensional normalized matrices from all 264 subjects’ data were concatenated by frame. Then, *k*-means clustering was performed to classify frames into different clusters based on their spatial similarity, and cluster centers were defined as TNSs. As in previous studies, clustering was performed with all subjects together instead of separating subjects into ASD and CON groups so that the features were comparable between the two groups (Kupis et al., 2020; Marshall et al., 2020). Some CAP studies found that pairs of TNSs would display “mirror” patterns—the two TNSs in a pair showed opposite patterns of activation (Huang, Zhang, Wu, Mashour, & Hudetz, 2020; Yang et al., 2021). To address this, the cluster number *k* ranged from 2 to 10 with a step length of 2. After clustering, each frame had an index representing which TNS it belonged to.

To maintain a good balance between richness and redundancy (Liu & Duyn, 2013b), the elbow method was used to choose the appropriate *k* number (Supporting Information Figure S1). We chose to use six TNSs, which converted the DMN, ECN, and SN into three pairs of “mirror” patterns, because the sum of the squared error decreased slowly when *k* was larger than 6. The TNSs were normalized (*Z* map) by dividing by the within-cluster standard deviation. The normalized TNSs spatial maps for other cluster number *k* were displayed in Supporting Information Figure S2. By comparing different thresholds (from 0.3 to 0.5; more details in Supporting Information Figure S3), the threshold of stable activation was set as 0.4 (−0.4 for deactivation) to highlight the stable regions and preserve complete brain networks.

### Spatial Stability of Six TNSs

To investigate the spatial stability of each TNS, we evaluated two parameters: the reproducibility among sites and the distance to the center of each frame. A stable TNS should be repeatable among different sites and show a short distance to the center. To study which regions were stable, individual-level stable activation rate (iSAR) was calculated.

#### Reproducibility among multisites.

To evaluate the reproducibility among sites, the CAP analysis was performed within every site, setting the clustering number *k* as 6. The TNSs obtained from each site were matched with TNSs obtained from all 264 subjects according to the spatial similarity as well as the cluster indexes. The spatial similarity of relevant TNSs between any two sites was calculated using the Pearson correlation coefficient.

#### Distance to the center of each TNS.

The distance to the center of each frame was defined as 1 − *r*, where *r* was the Pearson correlation coefficient between a frame and its cluster center. The mean distance to the center of each TNS was calculated within each subject for further statistical analyses.

#### Individual-level stable activation rate (iSAR).

To figure out which regions were steadily activated or deactivated in a TNS among subjects, individual-specific TNS was calculated for each subject as the average of frames having same index within a subject and then dividing it by the within-cluster standard deviation. Then, the normalized activation intensity of this individual-specific TNS was compared with the threshold of 0.4 (−0.4 for deactivation) for each region. Regions that had stronger activation or deactivation than threshold were considered activated or deactivated steadily. If a region was activated or deactivated steadily in more than 50% of the subjects, it was defined as a stable region. For all the stable regions of a TNS, the iSAR was calculated for each subject. The iSAR for one region was defined as *n*/*N*; *n* is the number of frames with stable activation (absolute value of intensity > 0.4), and *N* is the total frame number of this individual-specific TNS. Thus, the iSAR represents the proportion of steadily activated frames out of the total number of frames for that individual-specific TNS for a parcel.

### Temporal Features of Six TNSs

Using the indexes of frames for each subject, temporal features were calculated to evaluate the dynamic characteristics: (a) dwell time—the proportion of time spent in a TNS over the time series—and (b) transition probability among TNSs—the ratio of the number of transitions from one TNS to another to the number of total transitions.

### Statistical and Correlation Analysis

The site variability, TR value, and eye status (open or closed) were removed using the *ComBat* method (Fortin et al., 2018; Johnson, Li, & Rabinovic, 2007) before carrying out statistical analyses. A mixed-design three-way analysis of variance (ANOVA; 6 TNSs × ASD/CON × juveniles/adults) was performed to determine the effect of TNS, group, and age on distance to the center of each TNS and dwell times for all subjects. The TNS was the within-subject factor, and the group and age were the between-subject factors. Post hoc two-sample *t* tests were conducted to obtain pairwise comparison results. The full IQ and mean absolute motion were set as covariates. The false discovery rate (FDR) method was used to correct for multiple comparisons in this research. To verify the effects of TNSs and group on the distance to the center of each TNS, a two-way ANOVA (6 TNSs × ASD/CON) was also performed on the distance to the center of each frame within each site.

Two-sample *t* tests were performed on the iSAR values of each TNS and transition probability among TNSs to study the differences between juvenile ASD and CON groups and between adult ASD and CON groups, with age, full IQ, and mean absolute motion as covariates. The general linear model for *t* tests is listed in the Supporting Information.

In this study, the Social Responsiveness Scale (SRS) *T* scores of 5 items (awareness, Aware; cognition, Cogn; communication, Comm; motivation, Mot; mannerisms, Manner) and total *T* score were used to study the correlation between social deficits and brain features. Only subjects who had SRS records were included (65 juvenile ASD, 64 juvenile CON, 27 adult ASD, 28 adult CON). The age, full IQ, and mean absolute motion were regressed from the CAP features. The age, full IQ, and scale version were regressed from the SRS *T* scores.

To study the relationship between the iSAR and social deficits, canonical correlation analysis (CCA) (Hotelling, 1936) was performed. CCA is a multivariate statistical method to reveal the correlation between two sets of variables. In this case, CCA converted the iSAR values and SRS *T* scores into canonical variate (CV) pairs. The correlation between the first CV pair represented the correlation between iSAR values and SRS *T* scores. The significance was estimated via 10,000 permutations using the code from Smith et al. (2015). To interpret the correlation between iSAR values and SRS *T* scores, the canonical loadings were calculated as the Pearson correlation coefficients between the original features and its first CV, which reflects the degree to which an original variable is represented by its CV (Alpert & Peterson, 1972; Gu & Wu, 2018). A detailed explanation of the CCA method can be found in the Supporting Information.

To study the relationship between CAP temporal features and SRS *T* scores and verify the correlation between iSAR values and SRS *T* scores, the Pearson correlation coefficient between each temporal feature and each SRS item was calculated.

## RESULTS

### Six TNSs Shared by ASD and CON

**Figure 1.**
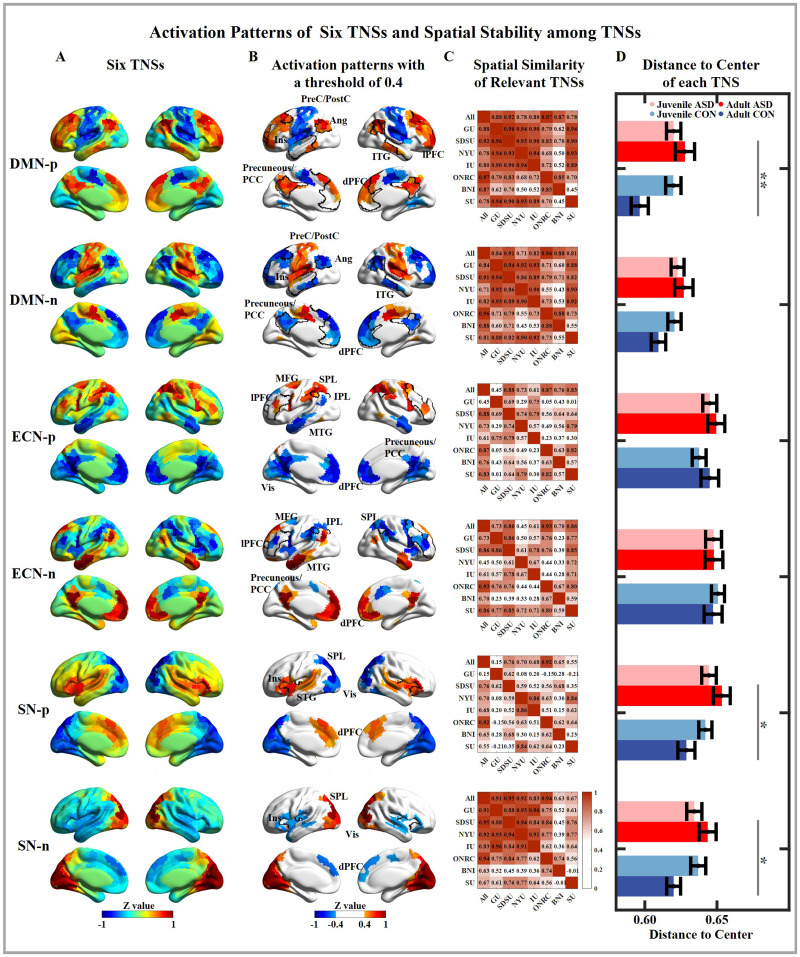
Activation patterns of six TNSs and spatial stability of TNSs. Each row represents one TNS. (A) The normalized spatial maps of the six TNSs. (B) The normalized spatial maps of the six TNSs with a threshold of 0.4. Regions showing stable activation are labeled with abbreviated region names, and hub regions of the dominating network are outlined with a black line. (C) Spatial similarity among relevant TNSs from all subjects and each site. (D) The group mean distance to the center of each TNS. The error bar represents the standard error. The pink, red, light blue, and dark blue bars represent the juvenile ASD, the adult ASD, the juvenile CON, and the adult CON groups, respectively. Ang, angular gyrus; PreC, precentral gyrus; PostC, postcentral gyrus; MTG, middle temporal gyrus; ITG, inferior temporal gyrus; STG, superior temporal gyrus; PCC, posterior cingulate gyrus; dPFC, dorsal prefrontal cortex; MFG, middle frontal gyrus; lPFC, lateral prefrontal cortex; IPL, inferior parietal lobe; SPL, superior parietal lobe; Ins, insula; Vis, the visual network. * indicates *p* < 0.05, ** indicates *p* < 0.01 (FDR corrected).

We defined six TNSs shared by ASD and CON groups; the activation patterns are shown in Figures 1A and 1B. Based on the identified activation patterns, six TNSs were divided into three TNS pairs. Each TNS pair represents the activation pattern of one network in the triple network model. The TNSs that activated a network were labeled p, while the TNSs that deactivated a network were labeled with an n (e.g., DMN-p and DMN-n).

The first pair of TNSs focused on the DMN. The DMN-p had a stable activated dorsal prefrontal cortex (dPFC), temporal regions, angular gyrus, precuneus, and posterior cingulate cortex (PCC), which are hub regions of the DMN. The lateral prefrontal cortex (lPFC) was also coactivated with the DMN. The SN and SM were deactivated in the DMN-p. The DMN-n showed the opposite activation pattern of the DMN-p. The DMN-TNSs represented the “anticorrelation” between the DMN and the SM.

The second pair of TNSs focused on the ECN. The ECN-p showed a strong activation in the ECN and DAN hub region, including the lPFC, inferior parietal lobe (IPL), middle frontal gyrus (MFG), and superior parietal lobe (SPL). The DMN was deactivated in the ECN-p. The ECN-n had an opposite activation pattern compared with the ECN-p. The ECN and DAN are known as “task positive” networks. Thus, the ECN-TNSs represented the “anticorrelation” between the “task positive” networks and the DMN.

The third pair of TNSs focused on the SN. The SN-p showed activation of the insula (hub region of the SN), dPFC, and temporal regions. The SPL and the Vis were significantly deactivated. The SN-n showed opposite patterns of the SN-p. The SN-TNSs represented the “anticorrelation” between the SN and Vis.

### Spatial Stability of Six TNSs

Two aspects of spatial stability were evaluated for each TNS: the reproducibility among sites and the distance to the center of each frame. The age-related differences between ASD and CON groups in spatial stability for each TNS were evaluated with the distance to the center of each frame and iSAR.

#### Reproducibility of six TNSs.

As shown in Figure 1C, the DMN-TNSs were the most reproducible, with higher spatial similarities among sites compared with others. The ECN-TNSs showed the lowest reproducibility. The normalized *Z* maps of the TNSs obtained from each site are displayed in Supporting Information Figure S4.

#### Effects of TNS, group, and age on the distance to the center of each frame.

Using a three-way ANOVA, we found the significant main effect of TNS (*F* = 33.538, *p* < 0.001) and group (*F* = 6.209, *p* = 0.013) and significant group × age interaction (*F* = 6.520, *p* = 0.011) on the distance to the center of each frame (Supporting Information Table S1). As shown in Figure 1D and Supporting Information Figure S5, the DMN-TNSs had significantly shorter distances to the center, while the ECN-TNSs had significantly longer distances to the center. This indicates that the DMN-TNSs were the most stable while the ECN-TNSs were the most unstable. Similar trends of distances to the center of each frame were seen at each site (Supporting Information Figure S6). The DMN-TNSs always showed shorter distances than others.

**Figure 2.**
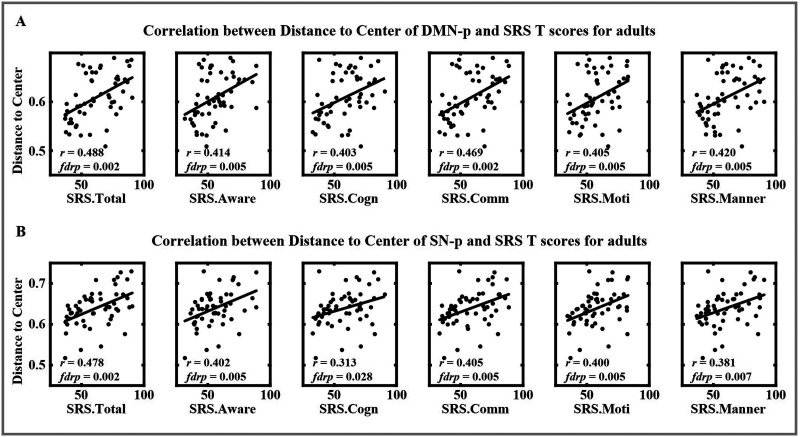
Correlation between the SRS T scores and the distance to the center of DMN-p and SN-p in adults. (A) Correlation between the distance to the center of DMN-p and SRS *T* scores in adults. (B) Correlation between the distance to the center of SN-p and SRS *T* scores in adults. Significant positive correlations implied that the increased distance to the center was related to severer social deficits in autistic adults.

Adults showed a lower distance to center than juveniles for CON groups in DMN-and SN-TNSs, especially in the DMN-p (*t* = −2.720, *p* = 0.007) and SN-n (*t* = −2.098, *p* = 0.037) (Figure 1D), but the differences did not survive after FDR correction. This indicates that the spatial stability of CAPs would increase with age in healthy CON. However, this trend was not found in ASD groups. For adults, a higher distance to the center in the adult ASD group was found compared with the adult CON group in DMN-p (*t* = 3.580, *p* < 0.001, *fdrp* = 0.005), SN-p (*t* = 2.906, *p* = 0.004, *fdrp* = 0.024), and SN-n (*t* = 2.695, *p* = 0.008, *fdrp* = 0.030). The distance to the center of DMN-p and SN-p had a significant correlation with SRS *T* scores in adults (Figure 2), indicating that the unstable TNSs in adults with ASD was related to their social deficits. There was no difference on the distance to the center between juvenile ASD and juvenile CON groups. When comparing the difference between ASD and CON groups within a single site, there is a significant difference between ASD and CON groups in three adult sites (IU, ONRC, and BNI) and NYU (Supporting Information Figure S6 and Supporting Information Table S2), but no difference survived after the FDR correction. Our results suggest an abnormal developmental trajectory of the spatial stability of transient states in people with ASD, especially on the DMN-p. The three-way ANOVA was also performed to the distance to the center under different cluster number *k*, which also confirmed this; see the pairwise comparisons of group and age on the distance to the center under different *k* numbers in Supporting Information Figure S7.

#### Age-related differences in iSAR between ASD and CON groups.

**Figure 3.**
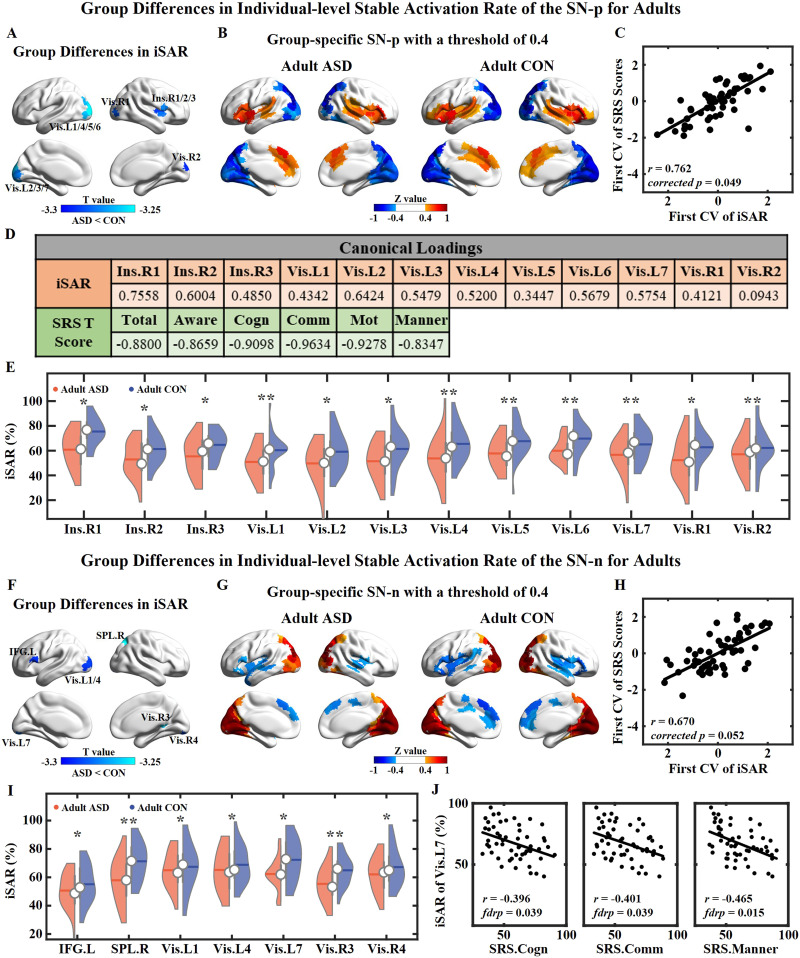
Group differences in iSAR for the SN-TNSs between adult ASD and CON groups. (A) Group difference map of iSAR values for the SN-p. Parcels of the right insula and the Vis show significantly decreased iSAR in the adult ASD group. (B) Group-specific normalized spatial maps of SN-p with a threshold of 0.4. (C) The correlation between the first pair of CVs. (D) Canonical loadings of original features and its first CV. (E) Violin plots of SN-p iSAR values for 12 parcels. (F) Group difference map of iSAR values for the SN-n. Parcels of the left IFC, the right SPL, and the Vis show significantly decreased iSAR in the adult ASD group. (G) Group-specific normalized spatial maps of SN-n with a threshold of 0.4. (H) The correlation between the first pair of CVs. (I) Violin plots of SN-n iSAR values for seven parcels. For (E) and (I), the red shape represents the adult ASD group, and the blue shape represents the adult CON group. The dark, horizontal lines within each shape show the mean value of the group; the white circles show the median of the group; and the vertical, dark gray bars represent the upper and lower quartile points. (J) Pearson correlations between the SN-n iSAR value of Vis.L7 and SRS *T* scores. *r* is the correlation coefficient, and *fdrp* is the FDR-corrected *p* value. * indicates *p* < 0.05, ** indicates *p* < 0.01 (FDR corrected).

For iSAR, the hub regions of the dominating network in each TNS showed a high iSAR value in four groups (Supporting Information Figure S8). There was no difference in iSAR between juvenile ASD and CON groups. Compared with the adult CON group, the adult ASD group showed lower iSAR on the SN-TNSs. For the SN-p, the adult ASD group showed lower iSAR on three parcels in the right insula and nine parcels in the Vis (Figures 3A and 3E); see the details of the coordinates in MNI space and two-sample *t* tests in Supporting Information Table S3. When calculating the centroid of SN-p within the adult ASD group and within the adult CON group severally (Figure 3B), the insula and the Vis showed weaker intensity in the adult ASD group, which indicates that these regions had more unstable activation/deactivation in the adult ASD group. Using the CCA method, we found a significant correlation between the SN-p iSAR of these 12 regions and SRS *T* scores based on the correlation coefficient of first CV pair (*r* = 0.762, *corrected p* = 0.049), with 27 adult ASD and 28 adult CON subjects (Figure 3C). The canonical loadings between the original features and the first pair of CVs are shown in Figure 3D. The iSAR canonical loadings were positive, while the SRS *T* canonical loadings were negative, indicating that the iSAR had a negative correlation with SRS *T* scores. The top five parcels with the highest canonical loading are located on right insula (Ins.R1, Ins.R2) and left occipital cortex (Vis.L2, Vis.L6, and Vis.L7), and each item of SRS *T* scores showed a high canonical loading (lower than −0.8). This indicates that the correlation between the iSAR of SN-p and SRS *T* scores mostly came from the negative correlation between the iSAR of the right insula and left occipital pole and SRS *T* scores. These results suggested that the decrease in spatial consistency of these regions was related to more severe social deficits. This was also verified through Pearson correlations between the iSAR values and each SRS *T* score. Except the Vis.R2, which had the lowest canonical loading, the SN-p iSAR values of the other 11 parcels showed a significant negative correlation with SRS items (Supporting Information Figures S9 and S10).

The adult ASD group also showed a lower SN-n iSAR in one parcel of the left inferior frontal gyrus (IFG.L), one parcel of the right SPL (SPL.R), and five parcels of the Vis (Figures 3F and 3I); see the details of the coordinates in MNI space and two-sample *t* tests in Supporting Information Table S4. As similar as in SN-p, in the centroid of SN-n within the adult ASD group, the insula, SPL, and Vis showed lower intensity than in the centroid of SN-n within the adult CON group. With CCA, no significant correlation was found between SN-n iSAR values and SRS *T* scores (Figure 3H). For the SN-n iSAR value of Vis.L7, significant negative correlations with SRS cognition *T* score, communication *T* score, and mannerisms *T* score were found.

### Temporal Features of TNSs

#### Effects of TNS, group, and age on the dwell time of six TNSs.

**Figure 4.**
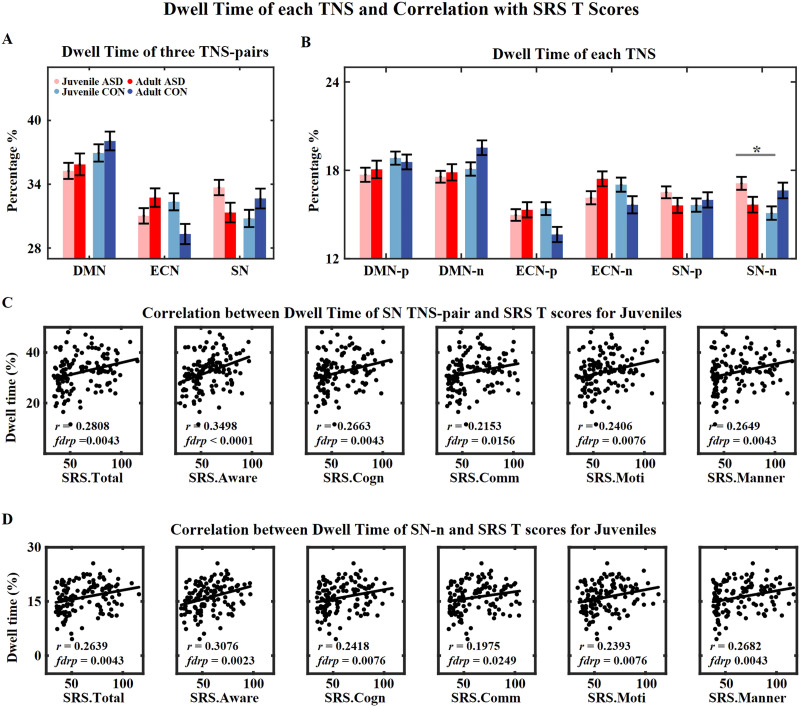
Dwell time of each TNS and correlation with SRS *T* scores. (A) Group mean dwell time of each TNS pair. The DMN-TNSs have the largest dwell time for all four groups. (B) Group mean dwell time of each TNS. For (A) and (B), the error bar represents the standard error. The pink, red, light blue, and dark blue bars represent the juvenile ASD, the adult ASD, the juvenile CON, and the adult CON groups, respectively. The juvenile ASD group has a larger dwell time for SN-TNSs than the juvenile CON group. The adult ASD group has a larger dwell time for ECN-TNSs than the adult CON group. (C) Correlations between dwell time of SN-TNS pair and SRS *T* scores for juveniles. (D) Correlations between dwell time of SN-n and SRS *T* scores for juveniles. *r* is the correlation coefficient, and *fdrp* is the FDR-corrected *p* value. * indicates *p* < 0.05 (FDR corrected).

Among the dwell time of three TNS pairs, we found the significant main effect of TNS (*F* = 28.444, *p* < 0.001) and the group × TNS × age interaction (*F* = 4.158, *p* = 0.016; Supporting Information Table S5). The DMN-TNSs had the longest dwell time for all four groups (Figure 4A; DMN-TNSs: juvenile ASD = 35.3%, adult ASD = 35.9%, juvenile CON = 36.9%, adult CON = 38.1%). Therefore, the DMN-TNSs were major states during resting state, while the ECN-TNSs and the SN-TNSs were minor states. See details of pairwise comparisons of TNSs for each group in Supporting Information Figure S11, and details of three-way ANOVA on six TNSs were listed in Supporting Information Table S6. For juveniles, the juvenile ASD group showed a higher dwell time for the SN-TNSs (*t* = 2.390, *p* = 0.018, *fdrp* = 0.052), especially SN-n (*t* = 3.013, *p* = 0.003, *fdrp* = 0.034). The dwell time of SN-TNSs was positively correlated with SRS *T* scores in juveniles, which indicated the increased dwell time of SN-TNSs in the juvenile ASD group related to severer social deficits (Figures 4C and 4D). For adults, the adult ASD group had a larger dwell time for ECN-TNSs (*t* = 2.579, *p* = 0.010, *fdrp* = 0.052). Overall, these results indicate that people with ASD spend less time on major states.

#### Transition probability among six TNSs.

**Figure 5.**
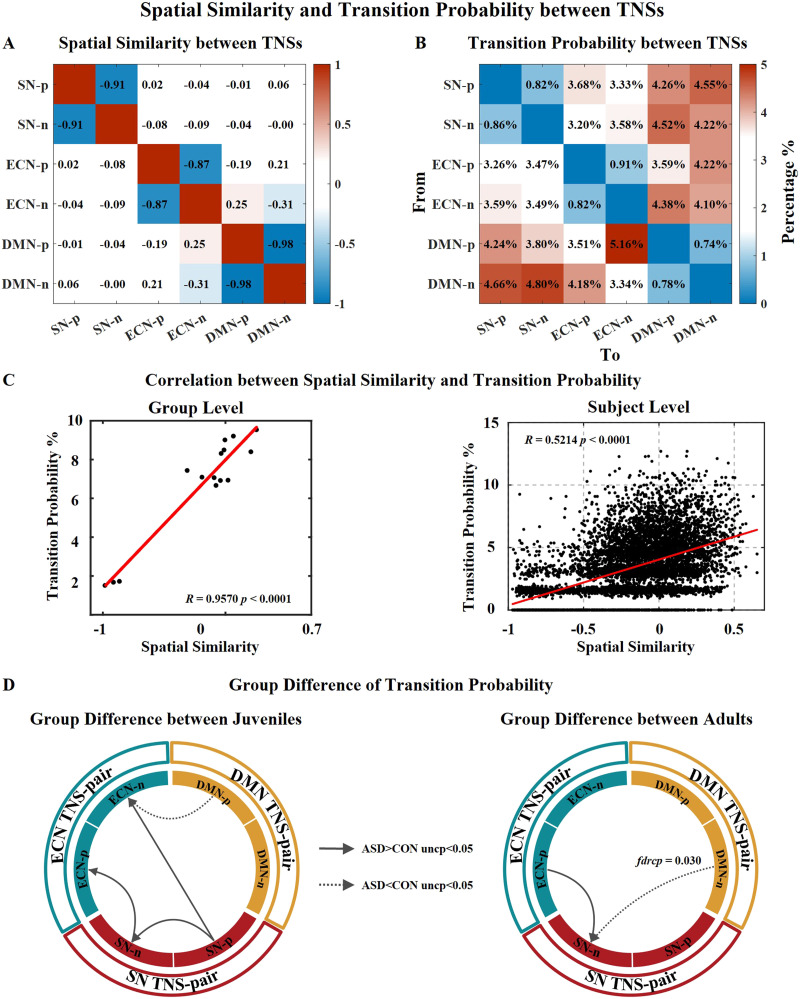
Spatial similarity and transition probability between TNSs. (A) Spatial similarity between any two TNSs. (B) Mean transition probability between TNSs of all 264 subjects. Transition probabilities between the DMN-TNSs and the SN-TNSs and between the DMN-TNSs and ECN-TNSs are relatively high, while the transitions probability between the ECN-TNSs and the SN-TNSs is relatively low. (C) Correlation between spatial similarity and transition probability. The group-level correlation is calculated with the normalized *Z* maps of the mean transition probability of all subjects. The individual-level correlation is calculated with the individual normalized *Z* maps of the transition probability for each subject. (D) Group differences in transition probability. The group differences are tested between the juvenile ASD and CON groups and between the adult ASD and CON groups severally. *uncp* is the uncorrected *p* value; *fdrp* is the FDR-corrected *p* value.

Transition probability was significantly correlated with spatial similarity among TNSs for all subjects at both the group level and the individual level, which is consistent with a previous study (Yang et al., 2021) (Figure 5C). There was almost no transition between paired TNSs since they show opposite patterns of activation.

Next, we examined internetwork transition probability (95.03% of all transitions). We found that the transition probability between the DMN-TNSs and SN-TNSs (35.05%) and between the DMN-TNSs and ECN-TNSs (32.48%) was higher compared with the transition probability between the SN-TNSs and ECN-TNSs (27.60%) in all subjects (Figure 5B and Supporting Information Figure S12). This indicates that the major transitions occurring within the triple network model during rest is related to the major states, the DMN-TNSs.

With two-sample *t* tests, we found that the juvenile ASD group had a higher transition probability from SN-p to ECN-n (*t* = 2.183, *p* = 0.015), from SN-p to SN-n (*t* = 1.812, *p* = 0.036), and from SN-n to ECN-p (*t* = 2.301, *p* = 0.011), while the group had a lower transition probability from DMN-p to ECN-n (*t* = −2.511, *p* = 0.007) than the juvenile CON group, but no difference survived after the FDR correction. For adults, the adult ASD group had a significantly lower transition probability from DMN-n to SN-n (*t* = −3.170, *p* = 0.001, *fdrp* = 0.030) and higher transition probability from ECN-p to SN-n (*t* = 1.933, *p* = 0.028). No significant correlation between transition probability and SRS *T* scores was found.

## DISCUSSION

In this work, we used the CAP analysis to study the spatial stability and temporal features of transient states in people with ASD. Six reliable TNSs were defined and divided into three pairs. The DMN-TNSs were major states during rest, which had the most stable spatial patterns and the longest dwell times, while the ECN-TNSs and SN-TNSs were minor states. We found age effects on the spatial stability; there was a trend of increased spatial stability of DMN-TNSs and SN-TNSs with age in healthy CON, but not in people with ASD. For the dwell times, autistic people tended to spend less time on major states and more time on minor states; autistic juveniles had a longer dwell time on the SN-TNSs while autistic adults had a longer dwell time on the ECN-TNSs. Although these results are obtained from male subjects with normal full IQ, we provided a new transient aspect for atypical brain functions in people with ASD.

### Spatial Stability and Temporal Features of Six TNSs

To our knowledge, this is the first study of spatial stability in functional TNSs on an individual level. Previous studies mainly focused on the reproducibility of TNSs among different datasets and preprocessing pipelines (Liu & Duyn, 2013a; Liu et al., 2018; Yang et al., 2021) without taking the differences in TNS spatial stability into account. Our results provide a transient-level explanation for the temporal variability of dFC from the view of spatial stability.

We found the DMN dominating major brain states during rest, with the longest dwell time and the most stable transient-level spatial patterns. These are in line with previous work showing that the DMN is the prominent network during rest (Raichle et al., 2001). In addition, the auditory and primary sensory regions that showed stable coactivation with the hub regions of DMN in the DMN-TNSs are known as “domain-specific” regions that perform specialized functions and are thought to be relatively modular (Fedorenko, Duncan, & Kanwisher, 2013; Power et al., 2011). These might be the reasons for the stable CAPs of the DMN-TNSs. Stable transient coactivation of these regions might lead to their stable connection on dFC, as reported to show lower temporal variability in healthy subjects in previous studies using traditional dFC (Yin et al., 2016; Zhang et al., 2016). Previous dFC studies also reported that the hub regions of the ECN had a higher temporal variability (Yin et al., 2016; Zhang et al., 2016). As for the brain function, the core regions of ECN (frontal and parietal regions), known as “domain-general” regions, are coactivated during a wide variety of cognitive tasks in a relatively diverse set of relationships, supporting cognitive flexibility (Crossley et al., 2013; Fedorenko et al., 2013; Power et al., 2011). Our result that ECN-TNSs showed a higher variance on spatial pattern was consistent with the higher temporal variability of ECN; both phenomena confirmed the general function of ECN.

Our results suggest that the transition probability might be influenced by the dwell time. Major internetwork transitions were related to major states, the DMN-TNSs, and minor transitions that occurred in minor states (between SN and ECN). Further, the transition proportion was correlated to the spatial similarity between TNSs, which is consistent with a previous report (Yang et al., 2021).

### Age-Related Unstable TNS Spatial Patterns in ASD

The TNSs represent coactivation/deactivation among brain networks, which indicates the functional integration/segregation among them. Previous connectome gradient studies suggest a clear functional segregation between the DMN and SM during rest, and the degree of segregation increased with age (Dong, Margulies, Zuo, & Holmes, 2021; Margulies et al., 2016; Xia et al., 2022). The major states in this work, DMN-TNSs, showed similar spatial patterns with the first connectome gradient, which indicated the consistency of transient states and the static connectome of the brain. We found that adults had more stable spatial patterns of DMN-TNSs in CON groups. This can be explained that as the brain matures, the degree of functional integration within networks and functional segregation among networks increases, leading to a more organized and efficient brain.

We observed differences in the spatial stability of TNSs primarily between the adult ASD and CON groups, rather than between juveniles. There was no trend of increasing spatial stability in ASD groups; the ASD adults even showed slightly more unstable DMN-TNSs and SN-TNSs than ASD juveniles. These results suggest lack of sufficient developmental changes in functional segregation with age among autistic people. The insufficient functional segregation in people with ASD has been reported before (Hong et al., 2019; Urchs et al., 2022). Higher temporal variability was also found in ASD based on dFC (Chen et al., 2017; Zhang et al., 2016). Xie et al. (2022) also reported higher modular variability in people with ASD. These results are consistent with ours. However, previous studies are based on mixed-age subjects. Our results are based on single scanning data from a multisite. It is necessary to further analyze the developmental trends of brain functional stability related to age in people with ASD based on longitudinal data with sufficient sample size in the future.

We found that the adult ASD group had significantly lower iSAR values of right insula and Vis for the SN-TNSs. The insula plays an important role in social communication; many studies find the atypical FC related to insula in ASD (Abbott et al., 2016; Hogeveen et al., 2018). Thus, the dysfunction of insula would lead to social deficits in people with ASD, as shown as the significant correlation between SN-p iSAR values of the right insula and SRS *T* scores in this study. The large variance of the Vis in adult ASD has also been reported before. Watanabe, Rees, and Masuda (2019) reported that adults with ASD showed more random activity of the inferior occipital gyrus during rest. The atypical function of the Vis in ASD might be related to its visual sensory symptoms (Simmons et al., 2009).

### Less Dwell Times of DMN-TNSs Between ASD and CON Groups

We found that the juvenile ASD group had a significantly larger dwell time for SN-TNSs than the juvenile CON group. While for adults, a larger dwell time for ECN-TNSs was found in the adult ASD group than the adult CON group. In the meanwhile, the dwell time of DMN-TNSs was relatively decreased in juvenile and adult ASD groups. As for transition proportion, the DMN-related transition was decreased in both juvenile and adult ASD groups, while the SN-related transition increased in the juvenile ASD group and the ECN-related transition increased in the adult ASD group, which might be influenced by the imbalanced activation proportion of TNSs in people with ASD. The ECN-TNSs showed the coactivation of ECN and DAN, which are thought to be related to external information (Menon & D’Esposito, 2022). The SN can evaluate the intensity and saliency of external stimuli and integrates this information to coordinate interactions (Menon, 2011; Uddin, 2015). Further, the parietal cortex was one of the hub regions of both ECN-TNSs and SN-TNSs, which plays important role in “bottom-up” attention driven by external salient stimuli (Buschman & Miller, 2007). Therefore, the ECN-TNSs and SN-TNSs may relate to the process of bottom-up attention during resting state. Our results indicated the abnormal function of bottom-up attention in people with ASD. People with ASD had an atypical balance characterized by enhanced low-level bottom-up processing and decreased top-down processing, leading to their increased focus on details and difficulties getting the “global picture” (Sapey-Triomphe et al., 2020). Amso, Haas, Tenenbaum, Markant, and Sheinkopf (2014) found that children with ASDs were more influenced by bottom-up visual scene information regardless of whether social stimuli and bottom-up scene properties were congruent or competing. Coderre et al. (2018) reported that adults with ASD would use a more bottom-up style of processing. These would explain the longer dwell time on the ECN-TNSs and SN-TNSs in people with ASD. Furthermore, the increased dwell time of SN-TNSs in the juvenile ASD group and increased dwell time of ECN-TNSs in the adult ASD group implied the different manifestations of attentional dysfunction in juveniles and adults with ASD, which is consistent with a previous FC study (Farrant & Uddin, 2016).

## ACKNOWLEDGMENTS

We want to thank Vinoo Alluri for useful comments on an earlier version of this manuscript.

## SUPPORTING INFORMATION

Supporting information for this article is available at https://doi.org/10.1162/netn_a_00396.

## AUTHOR CONTRIBUTIONS

Yunge Zhang: Conceptualization; Data curation; Formal analysis; Investigation; Software; Writing – original draft; Writing – review & editing. Lin Lin: Validation. Dongyue Zhou: Data curation. Yang Song: Writing – review & editing. Abigail Stein: Writing – review & editing. Shuqin Zhou: Writing – review & editing. Huashuai Xu: Validation. Wei Zhao: Validation. Fengyu Cong: Project administration; Supervision. Jin Sun: Project administration; Supervision; Validation. Huanjie Li: Conceptualization; Project administration; Supervision; Validation; Writing – review & editing. Fei Du: Project administration; Writing – review & editing.

## FUNDING INFORMATION

Huanjie Li, Science and Technology Planning Project of Liaoning Provincial, Award ID: 2022JH2/10700002. Fengyu Cong, Science and Technology Planning Project of Liaoning Provincial, Award ID: 2021JH1/10400049. STI 2030 - Major Projects, Award ID: 2022ZD0211500.

## DATA AVAILABILITY STATEMENT

The data that support the findings of this study are openly available in Autism Brain Imaging Data Exchange II dataset at https://fcon_1000.projects.nitrc.org/indi/abide/abide_II.html.

## Supplementary Material



## TECHNICAL TERMS

Dynamic functional connectivity: Functional connectivity within a short section rather than the whole time series, and sliding window is the commonly used approach.
Coactivation pattern analysis: Clustering-based method to calculate transient network states.
Transient network state: Common coactivation pattern with a single-frame resolution.
Dwell time: The proportion of time spent in one transient state over the time series.
Temporal variability: Variances of dynamic functional connectivity, usually calculated as a standard deviation among windows for each pair of connectivity.
Individual-level stable activation rate: The ratio of steadily activated frames to total frames for a region of single subject.
Transition probability: The ratio of transition number from one transient to another to the total transition number.
Canonical correlation analysis: A method to evaluate the relationship between two sets of variables rather than one-to-one relations (e.g., Pearson correlation).
Task positive networks: Networks that are involved in processing external oriented information and will be activated during tasks, like the ECN and DAN.

## References

[bib1] Abbott, A. E., Nair, A., Keown, C. L., Datko, M., Jahedi, A., Fishman, I., & Müller, R. A. (2016). Patterns of atypical functional connectivity and behavioral links in autism differ between default, salience, and executive networks. Cerebral Cortex, 26(10), 4034–4045. 10.1093/cercor/bhv191, 26351318 PMC5027998

[bib2] Allen, E. A., Damaraju, E., Plis, S. M., Erhardt, E. B., Eichele, T., & Calhoun, V. D. (2014). Tracking whole-brain connectivity dynamics in the resting state. Cerebral Cortex, 24(3), 663–676. 10.1093/cercor/bhs352, 23146964 PMC3920766

[bib3] Alpert, M. I., & Peterson, R. A. (1972). On the interpretation of canonical analysis. Journal of Marketing Research, 9(2), 187–192. 10.1177/002224377200900211

[bib4] Amso, D., Haas, S., Tenenbaum, E., Markant, J., & Sheinkopf, S. J. (2014). Bottom-up attention orienting in young children with autism. Journal of Autism and Developmental Disorders, 44(3), 664–673. 10.1007/s10803-013-1925-5, 23996226 PMC4089391

[bib5] Bi, X.-A., Zhao, J., Xu, Q., Sun, Q., & Wang, Z. (2018). Abnormal functional connectivity of resting state network detection based on linear ICA analysis in autism spectrum disorder. Frontiers in Physiology, 9, 475. 10.3389/fphys.2018.00475, 29867534 PMC5952255

[bib6] Buschman, T. J., & Miller, E. K. (2007). Top-down versus bottom-up control of attention in the prefrontal and posterior parietal cortices. Science, 315(5820), 1860–1862. 10.1126/science.1138071, 17395832

[bib7] Caballero, C., Mistry, S., & Torres, E. B. (2020). Age-dependent statistical changes of involuntary head motion signatures across autism and controls of the ABIDE repository. Frontiers in Integrative Neuroscience, 14, 23. 10.3389/fnint.2020.00023, 32625069 PMC7311771

[bib8] Calhoun, V. D., Miller, R., Pearlson, G., & Adalı, T. (2014). The chronnectome: Time-varying connectivity networks as the next frontier in fMRI data discovery. Neuron, 84(2), 262–274. 10.1016/j.neuron.2014.10.015, 25374354 PMC4372723

[bib9] Chen, H., Nomi, J. S., Uddin, L. Q., Duan, X., & Chen, H. (2017). Intrinsic functional connectivity variance and state-specific under-connectivity in autism. Human Brain Mapping, 38(11), 5740–5755. 10.1002/hbm.23764, 28792117 PMC5783325

[bib10] Coderre, E. L., Cohn, N., Slipher, S. K., Chernenok, M., Ledoux, K., & Gordon, B. (2018). Visual and linguistic narrative comprehension in autism spectrum disorders: Neural evidence for modality-independent impairments. Brain and Language, 186, 44–59. 10.1016/j.bandl.2018.09.001, 30216902

[bib11] Crossley, N. A., Mechelli, A., Vértes, P. E., Winton-Brown, T. T., Patel, A. X., Ginestet, C. E., … Bullmore, E. T. (2013). Cognitive relevance of the community structure of the human brain functional coactivation network. Proceedings of the National Academy of Sciences, 110(28), 11583–11588. 10.1073/pnas.1220826110, 23798414 PMC3710853

[bib12] Di Martino, A., O’Connor, D., Chen, B., Alaerts, K., Anderson, J. S., Assaf, M., … Milham, M. P. (2017). Enhancing studies of the connectome in autism using the autism brain imaging data exchange II. Scientific Data, 4, 170010. 10.1038/sdata.2017.10, 28291247 PMC5349246

[bib13] Dong, H.-M., Margulies, D. S., Zuo, X.-N., & Holmes, A. J. (2021). Shifting gradients of macroscale cortical organization mark the transition from childhood to adolescence. Proceedings of the National Academy of Sciences, 118(28), e2024448118. 10.1073/pnas.2024448118, 34260385 PMC8285909

[bib14] Farrant, K., & Uddin, L. Q. (2016). Atypical developmental of dorsal and ventral attention networks in autism. Developmental Science, 19(4), 550–563. 10.1111/desc.12359, 26613549

[bib15] Fedorenko, E., Duncan, J., & Kanwisher, N. (2013). Broad domain generality in focal regions of frontal and parietal cortex. Proceedings of the National Academy of Sciences, 110(41), 16616–16621. 10.1073/pnas.1315235110, 24062451 PMC3799302

[bib16] Fortin, J.-P., Cullen, N., Sheline, Y. I., Taylor, W. D., Aselcioglu, I., Cook, P. A., … Shinohara, R. T. (2018). Harmonization of cortical thickness measurements across scanners and sites. NeuroImage, 167, 104–120. 10.1016/j.neuroimage.2017.11.024, 29155184 PMC5845848

[bib17] Gu, F., & Wu, H. (2018). Simultaneous canonical correlation analysis with invariant canonical loadings. Behaviormetrika, 45, 111–132. 10.1007/s41237-017-0042-8

[bib18] Hogeveen, J., Krug, M. K., Elliott, M. V., & Solomon, M. (2018). Insula-retrosplenial cortex overconnectivity increases internalizing via reduced insight in autism. Biological Psychiatry, 84(4), 287–294. 10.1016/j.biopsych.2018.01.015, 29523413 PMC6067993

[bib19] Hong, S.-J., de Wael, R. V., Bethlehem, R. A. I., Lariviere, S., Paquola, C., Valk, S. L., … Bernhardt, B. C. (2019). Atypical functional connectome hierarchy in autism. Nature Communications, 10(1), 1022. 10.1038/s41467-019-08944-1, 30833582 PMC6399265

[bib20] Hotelling, H. (1936). Relations between two sets of variates. Biometrika, 28(3–4), 321–377. 10.1093/biomet/28.3-4.321

[bib21] Huang, Z., Zhang, J., Wu, J., Mashour, G. A., & Hudetz, A. G. (2020). Temporal circuit of macroscale dynamic brain activity supports human consciousness. Science Advances, 6(11), eaaz0087. 10.1126/sciadv.aaz0087, 32195349 PMC7065875

[bib22] Janes, A. C., Peechatka, A. L., Frederick, B. B., & Kaiser, R. H. (2020). Dynamic functioning of transient resting-state coactivation networks in the Human Connectome Project. Human Brain Mapping, 41(2), 373–387. 10.1002/hbm.24808, 31639271 PMC7268046

[bib23] Jia, H., Hu, X., & Deshpande, G. (2014). Behavioral relevance of the dynamics of the functional brain connectome. Brain Connectivity, 4(9), 741–759. 10.1089/brain.2014.0300, 25163490 PMC4238311

[bib24] Johnson, W. E., Li, C., & Rabinovic, A. (2007). Adjusting batch effects in microarray expression data using empirical Bayes methods. Biostatistics, 8(1), 118–127. 10.1093/biostatistics/kxj037, 16632515

[bib25] Kupis, L., Romero, C., Dirks, B., Hoang, S., Parladé, M. V., Beaumont, A. L., … Uddin, L. Q. (2020). Evoked and intrinsic brain network dynamics in children with autism spectrum disorder. NeuroImage: Clinical, 28, 102396. 10.1016/j.nicl.2020.102396, 32891039 PMC7479441

[bib26] Liang, X., Zou, Q., He, Y., & Yang, Y. (2016). Topologically reorganized connectivity architecture of default-mode, executive-control, and salience networks across working memory task loads. Cerebral Cortex, 26(4), 1501–1511. 10.1093/cercor/bhu316, 25596593 PMC4785946

[bib27] Liégeois, R., Li, J., Kong, R., Orban, C., Van De Ville, D., Ge, T., … Yeo, B. T. T. (2019). Resting brain dynamics at different timescales capture distinct aspects of human behavior. Nature Communications, 10(1), 2317. 10.1038/s41467-019-10317-7, 31127095 PMC6534566

[bib28] Liu, X., & Duyn, J. H. (2013a). Resting-state fMRI signal anti-correlation exists in absence of global signal regression. Proceedings of the 21st ISMRM Annual Meeting.

[bib29] Liu, X., & Duyn, J. H. (2013b). Time-varying functional network information extracted from brief instances of spontaneous brain activity. Proceedings of the National Academy of Sciences of the United States of America, 110(11), 4392–4397. 10.1073/pnas.1216856110, 23440216 PMC3600481

[bib30] Liu, X., Zhang, N., Chang, C., & Duyn, J. H. (2018). Co-activation patterns in resting-state fMRI signals. NeuroImage, 180(Pt B), 485–494. 10.1016/j.neuroimage.2018.01.041, 29355767 PMC6082734

[bib31] Margulies, D. S., Ghosh, S. S., Goulas, A., Falkiewicz, M., Huntenburg, J. M., Langs, G., … Smallwood, J. (2016). Situating the default-mode network along a principal gradient of macroscale cortical organization. Proceedings of the National Academy of Sciences, 113(44), 12574–12579. 10.1073/pnas.1608282113, 27791099 PMC5098630

[bib32] Marshall, E., Nomi, J. S., Dirks, B., Romero, C., Kupis, L., Chang, C., & Uddin, L. Q. (2020). Coactivation pattern analysis reveals altered salience network dynamics in children with autism spectrum disorder. Network Neuroscience, 4(4), 1219–1234. 10.1162/netn_a_00163, 33409437 PMC7781614

[bib33] Menon, V. (2011). Large-scale brain networks and psychopathology: A unifying triple network model. Trends in Cognitive Sciences, 15(10), 483–506. 10.1016/j.tics.2011.08.003, 21908230

[bib34] Menon, V., & D’Esposito, M. (2022). The role of PFC networks in cognitive control and executive function. Neuropsychopharmacology, 47(1), 90–103. 10.1038/s41386-021-01152-w, 34408276 PMC8616903

[bib35] Power, J. D., Cohen, A. L., Nelson, S. M., Wig, G. S., Barnes, K. A., Church, J. A., … Petersen, S. E. (2011). Functional network organization of the human brain. Neuron, 72(4), 665–678. 10.1016/j.neuron.2011.09.006, 22099467 PMC3222858

[bib36] Raichle, M. E., MacLeod, A. M., Snyder, A. Z., Powers, W. J., Gusnard, D. A., & Shulman, G. L. (2001). A default mode of brain function. Proceedings of the National Academy of Sciences, 98(2), 676–682. 10.1073/pnas.98.2.676, 11209064 PMC14647

[bib37] Sapey-Triomphe, L.-A., Boets, B., Van Eylen, L., Noens, I., Sunaert, S., Steyaert, J., & Wagemans, J. (2020). Ventral stream hierarchy underlying perceptual organization in adolescents with autism. NeuroImage: Clinical, 25, 102197. 10.1016/j.nicl.2020.102197, 32014827 PMC6997624

[bib38] Schaefer, A., Kong, R., Gordon, E. M., Laumann, T. O., Zuo, X.-N., Holmes, A. J., … Yeo, B. T. T. (2018). Local-global parcellation of the human cerebral cortex from intrinsic functional connectivity MRI. Cerebral Cortex, 28(9), 3095–3114. 10.1093/cercor/bhx179, 28981612 PMC6095216

[bib39] Sharma, S. R., Gonda, X., & Tarazi, F. I. (2018). Autism spectrum disorder: Classification, diagnosis and therapy. Pharmacology & Therapeutics, 190, 91–104. 10.1016/j.pharmthera.2018.05.007, 29763648

[bib40] Simmons, D. R., Robertson, A. E., McKay, L. S., Toal, E., McAleer, P., & Pollick, F. E. (2009). Vision in autism spectrum disorders. Vision Research, 49(22), 2705–2739. 10.1016/j.visres.2009.08.005, 19682485

[bib41] Smith, S. M., Jenkinson, M., Woolrich, M. W., Beckmann, C. F., Behrens, T. E. J., Johansen-Berg, H., … Matthews, P. M. (2004). Advances in functional and structural MR image analysis and implementation as FSL. NeuroImage, 23, S208–S219. 10.1016/j.neuroimage.2004.07.051, 15501092

[bib42] Smith, S. M., Nichols, T. E., Vidaurre, D., Winkler, A. M., Behrens, T. E. J., Glasser, M. F., … Miller, K. L. (2015). A positive-negative mode of population covariation links brain connectivity, demographics and behavior. Nature Neuroscience, 18(11), 1565–1567. 10.1038/nn.4125, 26414616 PMC4625579

[bib43] Uddin, L. Q. (2015). Salience processing and insular cortical function and dysfunction. Nature Reviews Neuroscience, 16(1), 55–61. 10.1038/nrn3857, 25406711

[bib44] Urchs, S. G. W., Tam, A., Orban, P., Moreau, C., Benhajali, Y., Nguyen, H. D., … Bellec, P. (2022). Functional connectivity subtypes associate robustly with ASD diagnosis. eLife, 11, e56257. 10.7554/eLife.56257, 36444973 PMC9708070

[bib45] Vergara, V. M., Mayer, A. R., Damaraju, E., & Calhoun, V. D. (2017). The effect of preprocessing in dynamic functional network connectivity used to classify mild traumatic brain injury. Brain and Behavior, 7(10), e00809. 10.1002/brb3.809, 29075569 PMC5651393

[bib46] Watanabe, T., Rees, G., & Masuda, N. (2019). Atypical intrinsic neural timescale in autism. eLife, 8, e42256. 10.7554/eLife.42256, 30717827 PMC6363380

[bib47] Woolrich, M. W., Ripley, B. D., Brady, M., & Smith, S. M. (2001). Temporal autocorrelation in univariate linear modeling of FMRI data. NeuroImage, 14(6), 1370–1386. 10.1006/nimg.2001.0931, 11707093

[bib48] Xia, Y., Xia, M., Liu, J., Liao, X., Lei, T., Liang, X., … He, Y. (2022). Development of functional connectome gradients during childhood and adolescence. Science Bulletin, 67(10), 1049–1061. 10.1016/j.scib.2022.01.002, 36546249

[bib49] Xie, Y., Xu, Z., Xia, M., Liu, J., Shou, X., Cui, Z., … He, Y. (2022). Alterations in connectome dynamics in autism spectrum disorder: A harmonized mega- and meta-analysis study using the autism brain imaging data exchange dataset. Biological Psychiatry, 91(11), 945–955. 10.1016/j.biopsych.2021.12.004, 35144804

[bib50] Yang, H., Zhang, H., Di, X., Wang, S., Meng, C., Tian, L., & Biswal, B. (2021). Reproducible coactivation patterns of functional brain networks reveal the aberrant dynamic state transition in schizophrenia. NeuroImage, 237, 118193. 10.1016/j.neuroimage.2021.118193, 34048900

[bib51] Yeo, B. T. T., Krienen, F. M., Sepulcre, J., Sabuncu, M. R., Lashkari, D., Hollinshead, M., … Buckner, R. L. (2011). The organization of the human cerebral cortex estimated by intrinsic functional connectivity. Journal of Neurophysiology, 106(3), 1125–1165. 10.1152/jn.00338.2011, 21653723 PMC3174820

[bib52] Yin, D., Liu, W., Zeljic, K., Wang, Z., Lv, Q., Fan, M., … Wang, Z. (2016). Dissociable changes of frontal and parietal cortices in inherent functional flexibility across the human life span. Journal of Neuroscience, 36(39), 10060–10074. 10.1523/JNEUROSCI.1476-16.2016, 27683903 PMC6705576

[bib53] Zhang, J., Cheng, W., Liu, Z., Zhang, K., Lei, X., Yao, Y., … Feng, J. (2016). Neural, electrophysiological and anatomical basis of brain-network variability and its characteristic changes in mental disorders. Brain, 139(8), 2307–2321. 10.1093/brain/aww143, 27421791

