# Supplementary material for: Age-related unstable transient states and imbalanced activation proportion of brain networks in people with autism spectrum disorder: A resting-state fMRI study using coactivation pattern analyses
Source: Netw Neurosci. 2024 Dec 10;8(4):1173–91. doi: 10.1162/netn_a_00396 (PMC11674577; doi:10.1162/netn_a_00396)
Supplement: Supplementary file 1 [file netn-8-4-1173-s001.pdf]

Age-related unstable transient states and imbalance activation proportion of brain networks in people with autism spectrum disorder: resting-state fMRI study using co-activation pattern analyses

Clustering algorithm

We used k-means method to apply the co-activation pattern analysis. We repeated the clustering algorithm 100 times with a new initial cluster centroid for each k value, and we selected the results with the lowest sum of within-cluster point-to-center distances. The maximum number of iterations was 1000. The distance between one frame and its cluster center was defined as 1-r, where r was the Pearson correlation coefficient. The elbow method was used to choose appropriate k number. The sum of squared errors (SSE, within-cluster point-to-center distances) was calculated, when k was larger than six, the SSE decreased slowly, so we chose six as the appropriate k number. See the elbow plot in Fig. S1. The normalized spatial maps of TNSs for cluster number k of 2, 4, 8 and 10 were displayed in Fig.S2.

Threshold of stable activation on group-level

We used a set of thresholds on the normalized spatial maps of TNSs and finally found a threshold of 0.4 could highlight the stable regions and preserve complete brain networks. The spatial maps of TNSs under a threshold of 0.3, 0.4 and 0.5 were displayed in Fig.S3.

Three-way ANOVA

For distance to center of each frame and dwell times, three-way ANOVA (6 TNSs × ASD/CON × juveniles/adults) was performed to evaluate the effects of TNS, group and age. For post hoc t-tests, two-sample t-tests were performed to get pairwise comparison results and corrected with false discovery rate (FDR) method. The differences among TNSs were displayed in four groups severally as Fig.S5. Group differences between juvenile ASD and CON groups, between adult ASD and CON groups, between juvenile and adult ASD and between juvenile and adult with ASD at every TNS were displayed in Fig.1. To verify the effects of TNSs and group on the distance to the center of each frame, a two-way ANOVA (6 TNSs × ASD/CON) was also performed on distance to the center of each frame within each site (Fig.S6).

Two-sample t-tests

For group differences on the iSAR values of each TNS and transition probability among TNSs, the model was designed as (1), age, full IQ and mean absolute motion were covariates to remove the effects of them.

$$Y = \beta 0 + \beta 1 Group + \beta 2 IQ + \beta 3 Age + \beta 4 Motion + \varepsilon \quad (1)$$

Canonical correlation analysis

To study the relationship between individual-level stable activation rate (iSAR) and social deficits, canonical correlation analysis (CCA) was performed. For CCA analysis, X was an n-by-d1 matrix, Y was an n-by-d2 matrix, X and Y were the two feature

matrices, n was the number of subjects, d1 and d2 were number of features. The canonical variates (CVs) of the X and Y are defined as

$$U_i = Xa_i \quad (2)$$

$$V_i = Yb_i \quad (3)$$

where a_i and b_i maximize the Pearson correlation coefficient $\rho(U_i,V_i)$. In this work, X represented the iSAR values of regions showing group differences; Y represented SRS T scores. The significance level of CCA was evaluated via 10000 permutations of the rows of iSAR values. CCA was re-run after each permutation building up a valid null distribution of CCA results. The Pearson correlation coefficient of first pair of CVs (U_1 and V_1) represented the relationship of activation intensity and social deficits. The canonical loadings (CLs) of X and first CV of X (U_1) were defined as the Pearson correlation coefficients between U_1 and each column of X. The CLs of Y and first CV of Y (V_1) were defined as the Pearson correlation coefficients between V_1 and each column of Y. The CLs represented the relationship between original features and its CV.

Table S1. Three-way ANOVA on distance to center of each TNS.

**All subjects**	**Source**	**MS**	**MSE**	**df1**	**df2**	**F**	**Prob>F**
	Group	0.027	0.004	1	258	6.209	0.013
	Age	0.000	0.004	1	258	0.000	0.985
	Full IQ	0.000	0.004	1	258	0.068	0.795
	Mean absolute motion	0.041	0.004	1	258	9.245	0.003
	Group × Age	0.029	0.004	1	258	6.520	0.011
	TNS	0.046	0.001	5	1290	33.538	< 0.001
	Group × TNS	0.002	0.001	5	1290	1.734	0.124
	TNS × Age	0.002	0.001	5	1290	1.113	0.352
	Iq × TNS	0.002	0.001	5	1290	1.788	0.112
	Mean absolute motion ×	0.004	0.001	5	1290	2.614	0.023
	Group × TNS × Age	0.003	0.001	5	1290	1.829	0.104

MS, Mean square; MSE, Mean square error; df, Degrees of freedom

Table S2. Two-way ANOVA on distance to center of each frame within each site.

**GU**	**Source**	**MS**	**MSE**	**df1**	**df2**	**F**	**Prob>F**
	Group	0.000	0.007	1	36	0.072	0.790
	Full IQ	0.001	0.007	1	36	0.220	0.642
	Mean absolute motion	0.003	0.007	1	36	0.438	0.512
	TNS	0.004	0.001	5	180	3.103	0.010
	Group × TNS	0.002	0.001	5	180	1.539	0.180
	IQ × TNS	0.001	0.001	5	180	0.939	0.457
	Mean absolute motion ×	0.002	0.001	5	180	1.474	0.201
**SDSU**	**Source**	**MS**	**MSE**	**df1**	**df2**	**F**	**Prob>F**
	Group	0.007	0.005	1	38	1.334	0.255
	Full IQ	0.011	0.005	1	38	2.208	0.146
	Mean absolute motion	0.014	0.005	1	38	2.825	0.101
	TNS	0.010	0.002	5	190	6.271	< 0.001
	Group × TNS	0.001	0.002	5	190	0.545	0.742
	IQ × TNS	0.003	0.002	5	190	1.847	0.106
	Mean absolute motion ×	0.001	0.002	5	190	0.472	0.797
**NYU**	**Source**	**MS**	**MSE**	**df1**	**df2**	**F**	**Prob>F**
	Group	0.000	0.004	1	44	0.030	0.864
	Full IQ	0.000	0.004	1	44	0.031	0.860
	Mean absolute motion	0.010	0.004	1	44	2.307	0.136
	TNS	0.012	0.001	5	220	8.703	< 0.001
	Group × TNS	0.003	0.001	5	220	1.833	0.108
	IQ × TNS	0.001	0.001	5	220	0.990	0.424
	Mean absolute motion ×	0.001	0.001	5	220	0.866	0.504
**SU**	**Source**	**MS**	**MSE**	**df1**	**df2**	**F**	**Prob>F**
	Group	0.000	0.002	1	22	0.167	0.687
	Full IQ	0.000	0.002	1	22	0.071	0.792
	Mean absolute motion	0.000	0.002	1	22	0.000	0.988
	TNS	0.007	0.001	5	110	4.648	< 0.001
	Group × TNS	0.004	0.001	5	110	2.527	0.033
	IQ × TNS	0.001	0.001	5	110	0.615	0.689
	Mean absolute motion ×	0.004	0.001	5	110	2.931	0.016
**IU**	**Source**	**MS**	**MSE**	**df1**	**df2**	**F**	**Prob>F**
	Group	0.045	0.003	1	22	17.743	< 0.001
	Full IQ	0.003	0.003	1	22	1.001	0.328
	Mean absolute motion	0.006	0.003	1	22	2.451	0.132
	TNS	0.004	0.001	5	110	2.796	0.020
	Group × TNS	0.000	0.001	5	110	0.245	0.942
	IQ × TNS	0.001	0.001	5	110	0.995	0.425
	Mean absolute motion ×	0.001	0.001	5	110	0.891	0.490
**ONRC**	**Source**	**MS**	**MSE**	**df1**	**df2**	**F**	**Prob>F**
	Group	0.005	0.005	1	22	1.072	0.312
	Full IQ	0.000	0.005	1	22	0.042	0.840
	Mean absolute motion	0.006	0.005	1	22	1.286	0.269
	TNS	0.006	0.001	5	110	4.839	< 0.001
	Group × TNS	0.005	0.001	5	110	3.881	0.003

	IQ × TNS	0.002	0.001	5	110	1.797	0.119
	Mean absolute motion ×	0.001	0.001	5	110	0.607	0.695
**BNI**	**Source**	**MS**	**MSE**	**df1**	**df2**	**F**	**Prob>F**
	Group	0.013	0.006	1	52	1.980	0.165
	Full IQ	0.000	0.006	1	52	0.046	0.830
	Mean absolute motion	0.035	0.006	1	52	5.519	0.023
	TNS	0.016	0.001	5	260	11.981	< 0.001
	Group × TNS	0.002	0.001	5	260	1.790	0.115
	IQ × TNS	0.003	0.001	5	260	2.449	0.034
	Mean absolute motion ×	0.003	0.001	5	260	2.142	0.061

MS, Mean square; MSE, Mean square error; df, Degrees of freedom; GU, Georgetown University; SDSU, San Diego State University; NYU, New York University Langone Medical Center; SU, Stanford University; IU, Indiana University; ONRC, Olin Neuropsychiatry Research Center, Institute of Living at Hartford Hospital; BNI, Barrow Neurological Institute

Table S3. Two sample t tests of SN-p iSAR between adult ASD and CON groups

Parcel	MNI coordinates	T value<br>(adult ASD – adult CON)	P value after<br>FDR correction
Ins.L1	[40, 8, -2]	-3.170	0.017
Ins.L2	[40, -2, 6]	-2.845	0.038
Ins.L3	[54, 12, 12]	-3.291	0.014
Vis.L1	[-42, -86, -4]	-3.620	0.009
Vis.L2	[-8, -98, -8]	-3.377	0.013
Vis.L3	[-4, -88, 2]	-3.263	0.014
Vis.L4	[-24, -96, 6]	-4.096	0.007
Vis.L5	[-10, -96, 18]	-3.766	0.009
Vis.L6	[-26, -84, 22]	-3.566	0.009
Vis.L7	[-16, -86, -16]	-3.578	0.009
Vis.R1	[36, -88, 2]	-3.064	0.022
Vis.L2	[12, -94, 18]	-2.749	0.045

Table S4. Two sample t tests of SN-n iSAR between adult ASD and CON groups

Parcel	MNI coordinates	T value<br>(adult ASD – adult CON)	P value after<br>FDR correction
IFG.L	[-54, 20, 12]	-2.805	0.049
SPL.R	[20, -68, 52]	-4.017	0.006

Vis,L1	[-42, -86, -4]	-2.827	0.049
Vis.L4	[-24, -96, 6]	-2.883	0.049
Vis.L7	[-16, -86, -16]	-3.225	0.032
Vis.R3	[14, -46, 4]	-3.763	0.008
Vis.R4	[20, -88, -12]	-2.987	0.049

Table S5. Three-way ANOVA on dwell times of three TNS pairs.

**All subjects**	**Source**	**MS**	**MSE**	**df1**	**df2**	**F**	**Prob>F**
	Group	0.000	0.000	1	258	2.097	0.149
	Age	0.000	0.000	1	258	0.017	0.895
	Full IQ	0.000	0.000	1	258	2.847	0.091
	Mean absolute motion	0.000	0.000	1	258	2.094	0.149
	Group × Age	0.000	0.000	1	258	0.043	0.836
	TNS	0.195	0.007	2	516	28.444	< 0.001
	Group × TNS	0.015	0.007	2	516	2.222	0.109
	TNS × Age	0.003	0.007	2	516	0.491	0.612
	Iq × TNS	0.002	0.007	2	516	0.360	0.698
	Mean absolute motion ×	0.046	0.007	2	516	6.754	0.001
	Group × TNS × Age	0.029	0.007	2	516	4.158	0.016

MS, Mean square; MSE, Mean square error; df, Degrees of freedom

Table S6. Three-way ANOVA on dwell times of six TNSs.

**All subjects**	**Source**	**MS**	**MSE**	**df1**	**df2**	**F**	**Prob>F**
	Group	0.000	0.000	1	258	2.038	0.155
	Age	0.000	0.000	1	258	0.272	0.603
	Full IQ	0.000	0.000	1	258	0.546	0.461
	Mean absolute motion	0.000	0.000	1	258	1.186	0.277
	Group × Age	0.000	0.000	1	258	0.012	0.912
	TNS	0.047	0.002	5	1290	26.167	< 0.001
	Group × TNS	0.003	0.002	5	1290	1.757	0.119
	TNS × Age	0.002	0.002	5	1290	0.868	0.502
	Iq × TNS	0.001	0.002	5	1290	0.450	0.814
	Mean absolute motion ×	0.011	0.002	5	1290	6.182	< 0.001
	Group × TNS × Age	0.007	0.002	5	1290	3.759	0.002

MS, Mean square; MSE, Mean square error; df, Degrees of freedom

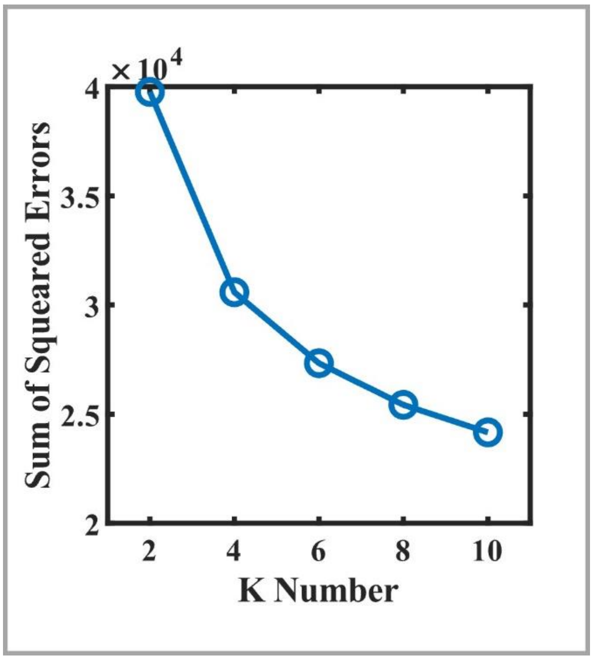

sFig. 1. Sum of squared errors for each k number. When k was larger than six, the SSE decreased slowly, so we chose six as the appropriate k number.

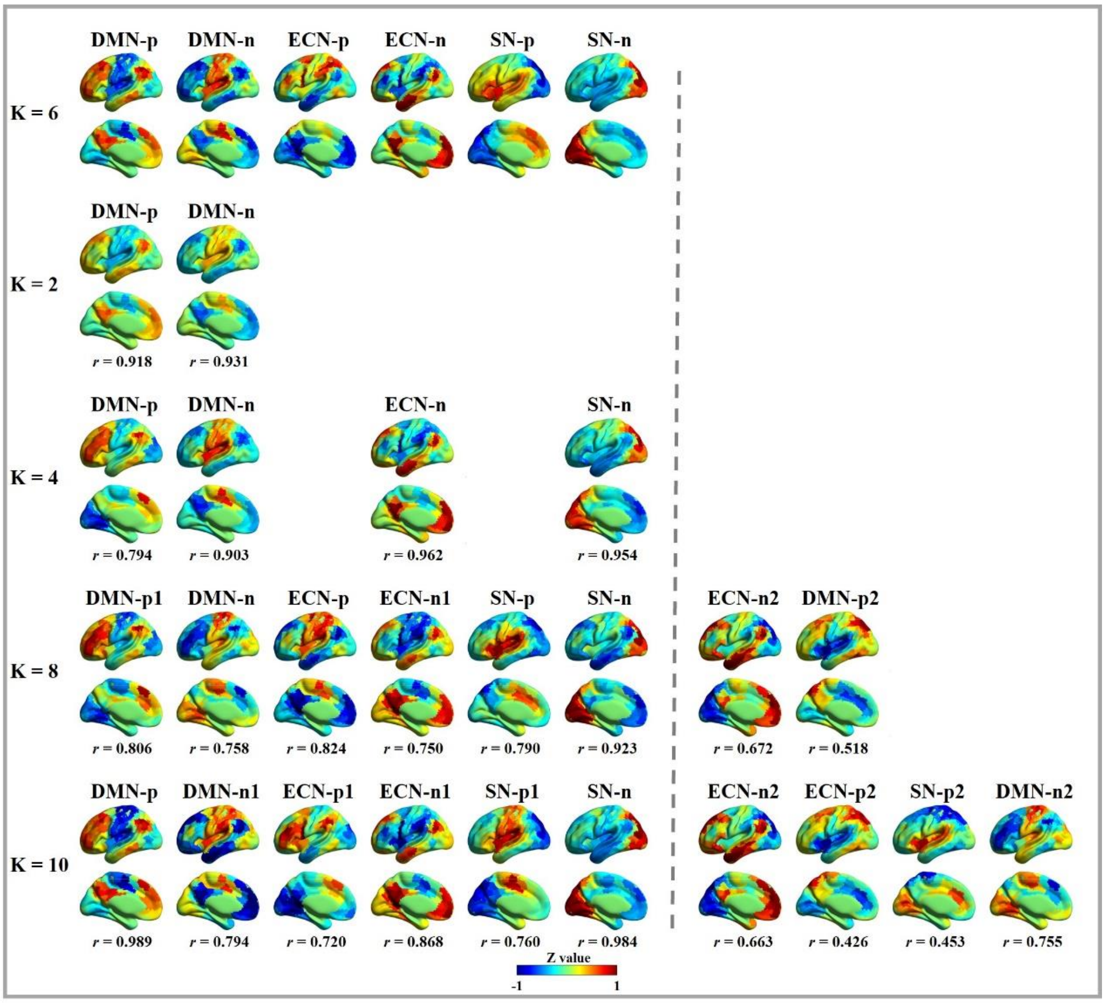

sFig. 2. Activation patterns of TNSs for different cluster number. Taking the results of cluster number 6 as a reference, we calculated the spatial similarity between the reference and results under other cluster numbers and named each TNS according to it. For results with cluster numbers of 8, and 10, the TNSs most similar to the reference are shown to the left of the dashed line, and the remaining states are shown to the right. *r* is the correlation coefficient between a TNS and its reference.

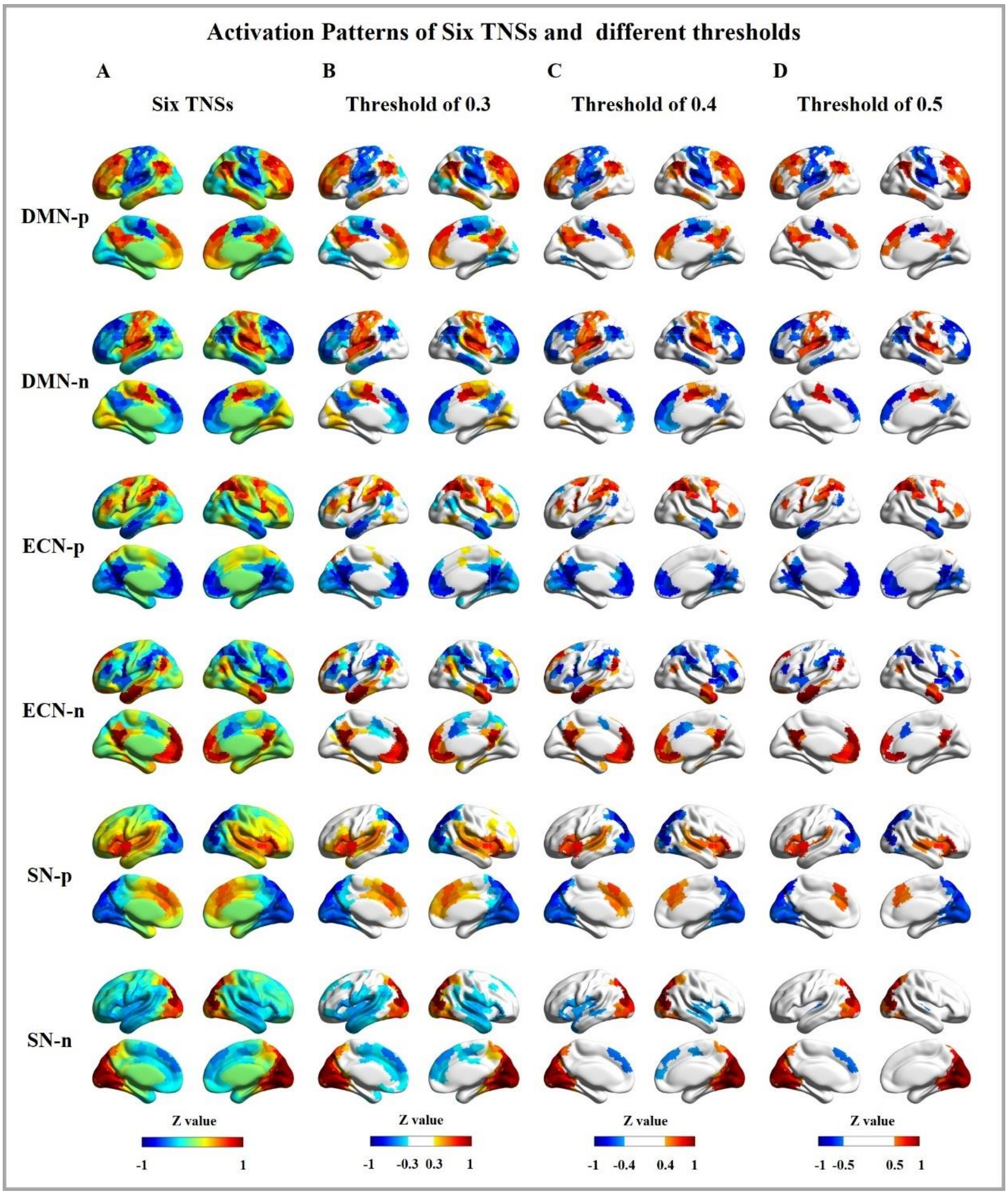

sFig. 3. Activation patterns of six TNSs and different thresholds. (A) Normalized spatial maps of six TNSs. (B) Normalized spatial maps with a threshold of 0.3. (C) Normalized spatial maps with a threshold of 0.4. (D) Normalized spatial maps with a threshold of 0.5.

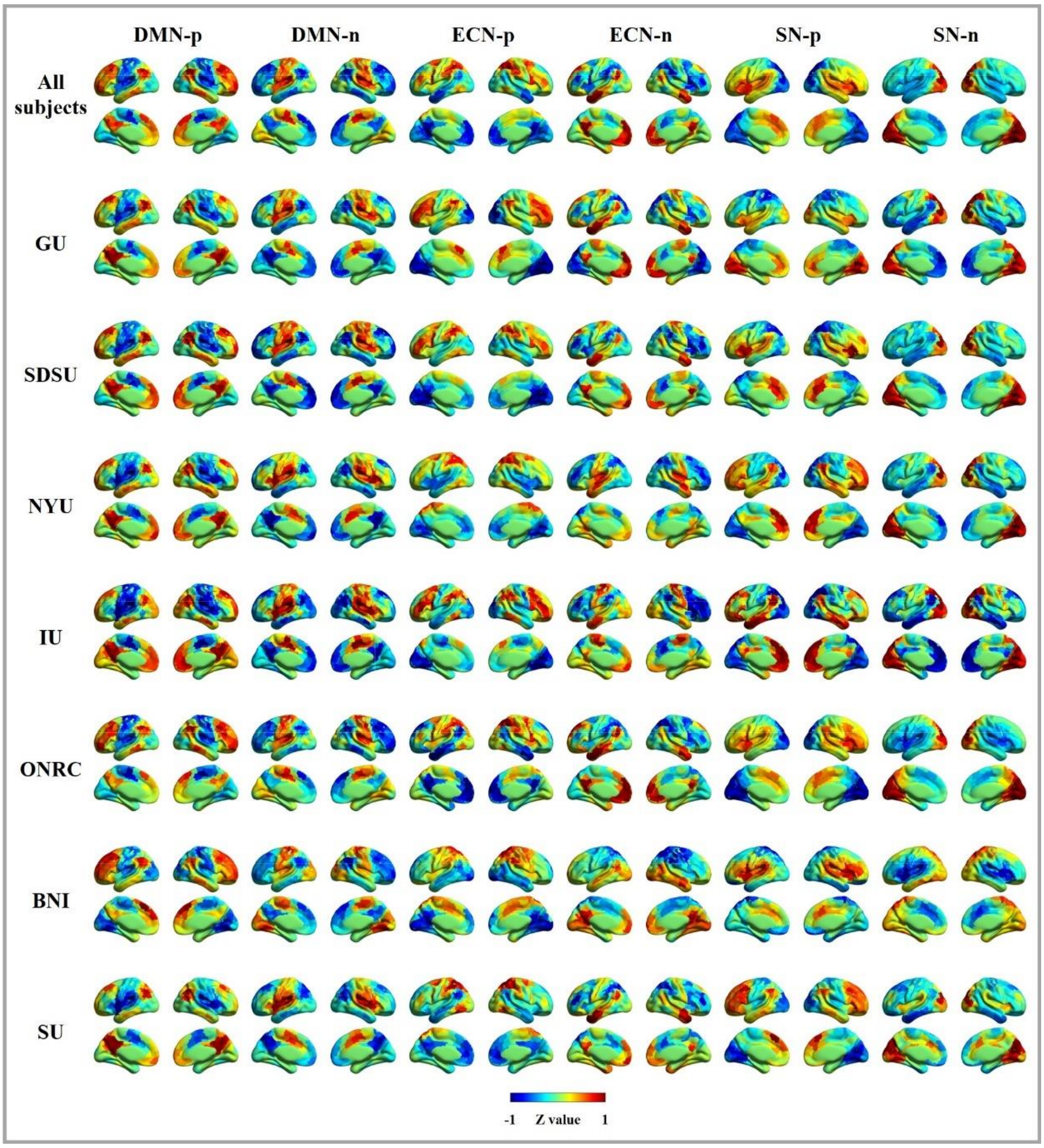

sFig. 4. Normalized Z maps of TNSs obtained from all subjects and obtained from each site.

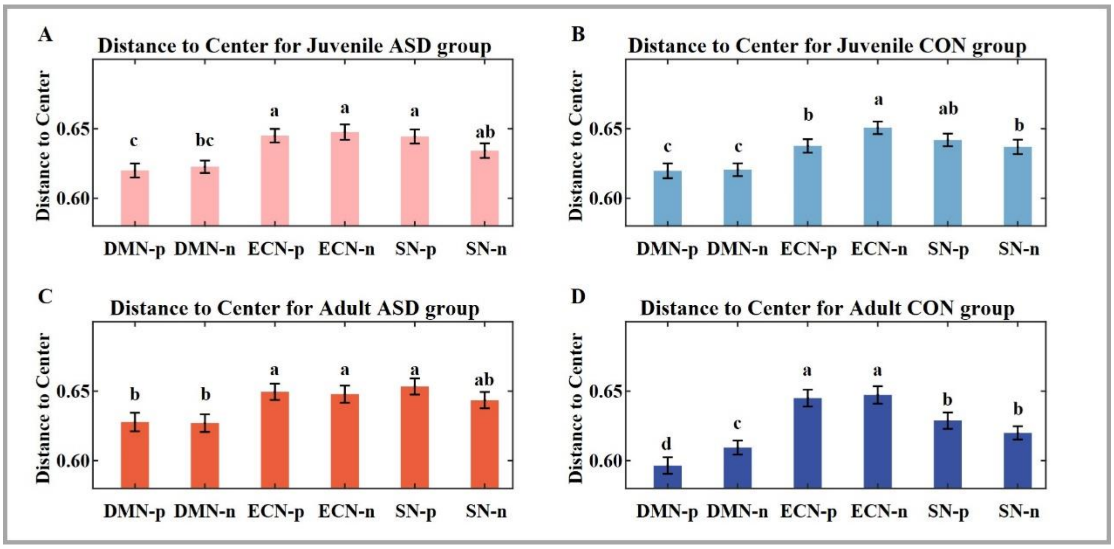

sFig. 5. Pairwise comparisons of TNS on distance to center for each group. The error-bar is standard error. TNSs have same labels means no significant difference (FDR corrected $p > 0.05$) between them. For example, in juvenile ASD group, 'ECN-n' and 'SN-n' both had label 'a', there is no difference between them.

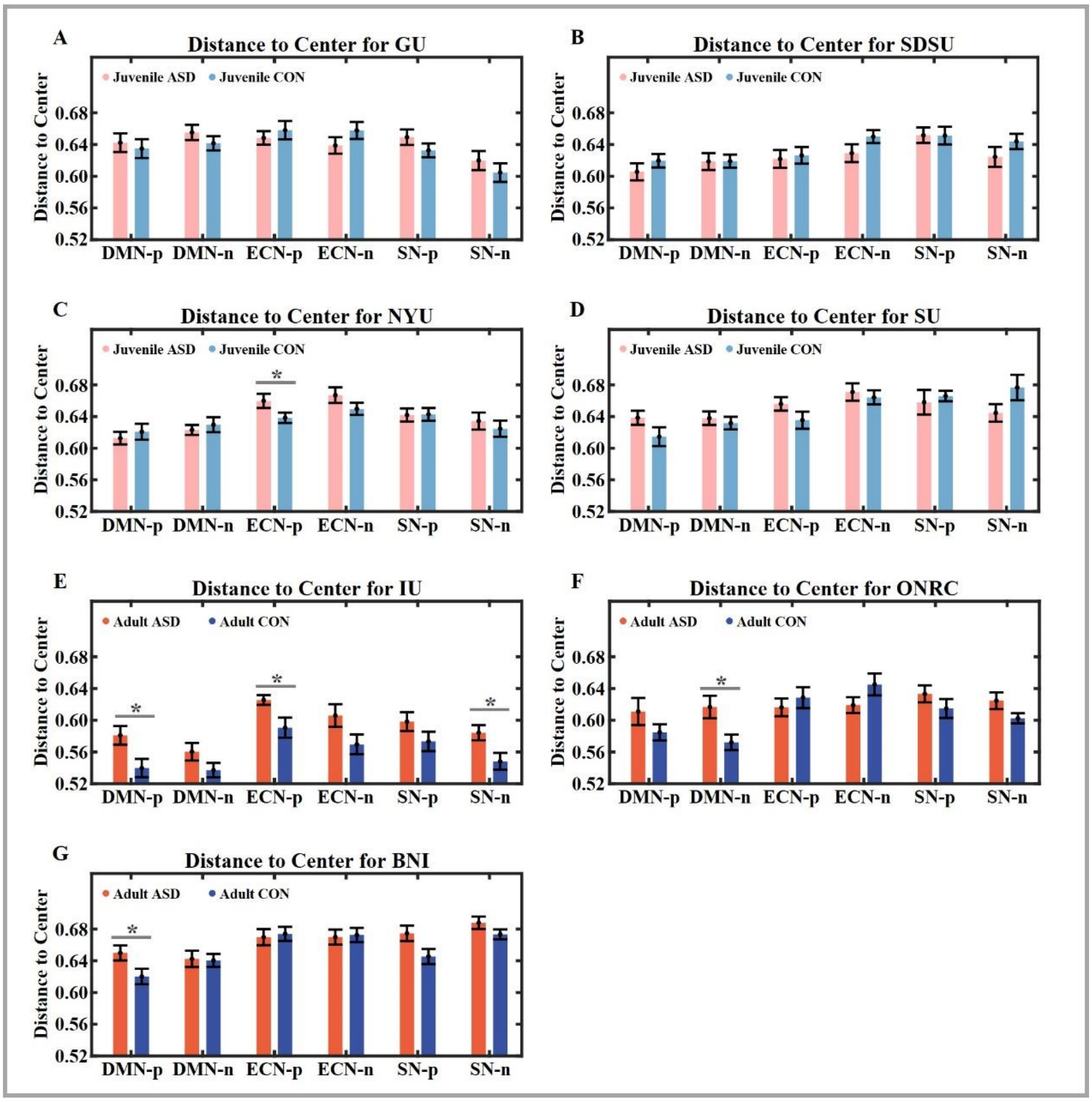

sFig. 6. Distance to center of each time point in each site. The error-bar is standard error. The distance is calculated with results obtained from all subjects and then displayed in each site. * indicates $p < 0.05$.

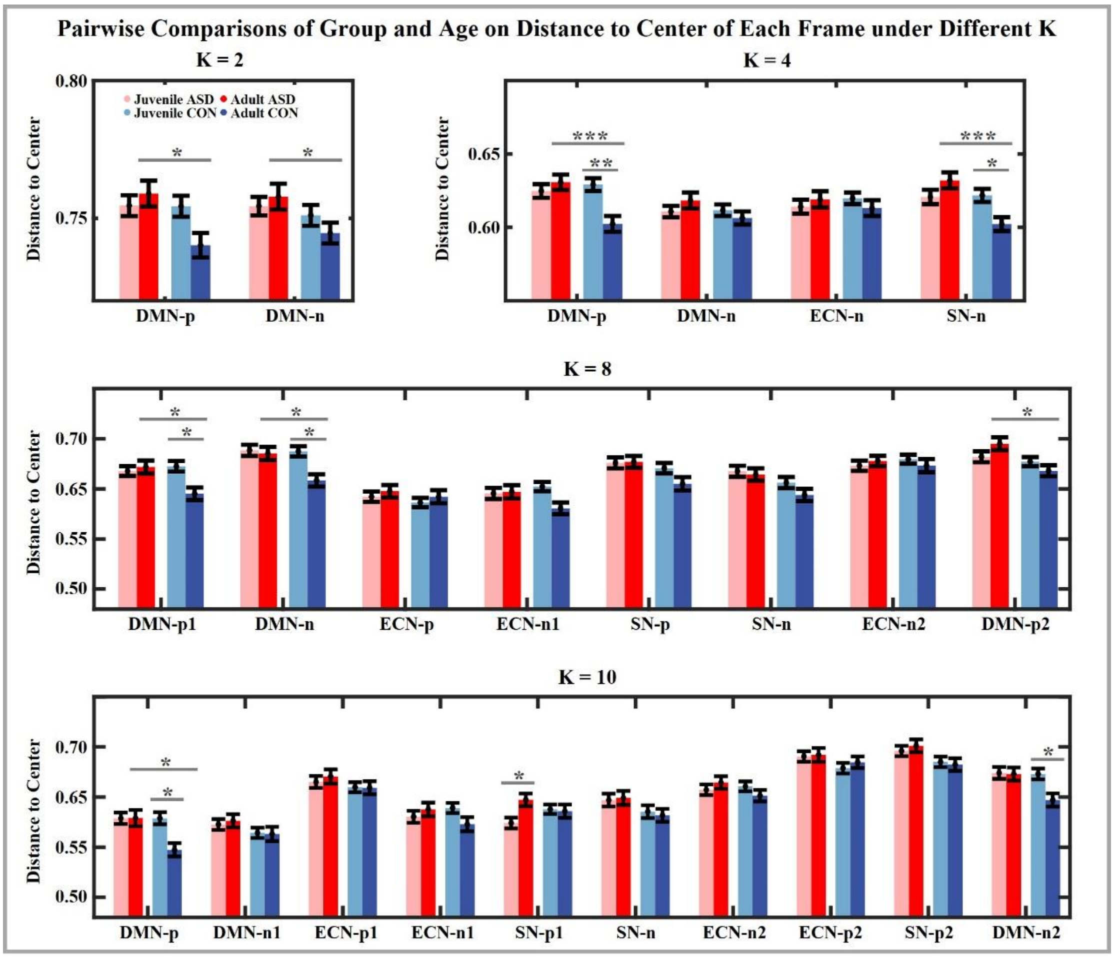

sFig. 7. Pairwise comparisons of group and age on distance to center of each frame under different k. For all the k number, there is a trend of decreased distance to center with age in both ASD groups and CON groups, and the distance to center decreased more with age in CON groups than in ASD groups. * indicates $p < 0.05$, ** indicates $p < 0.01$, *** indicates $p < 0.001$ (FDR corrected).

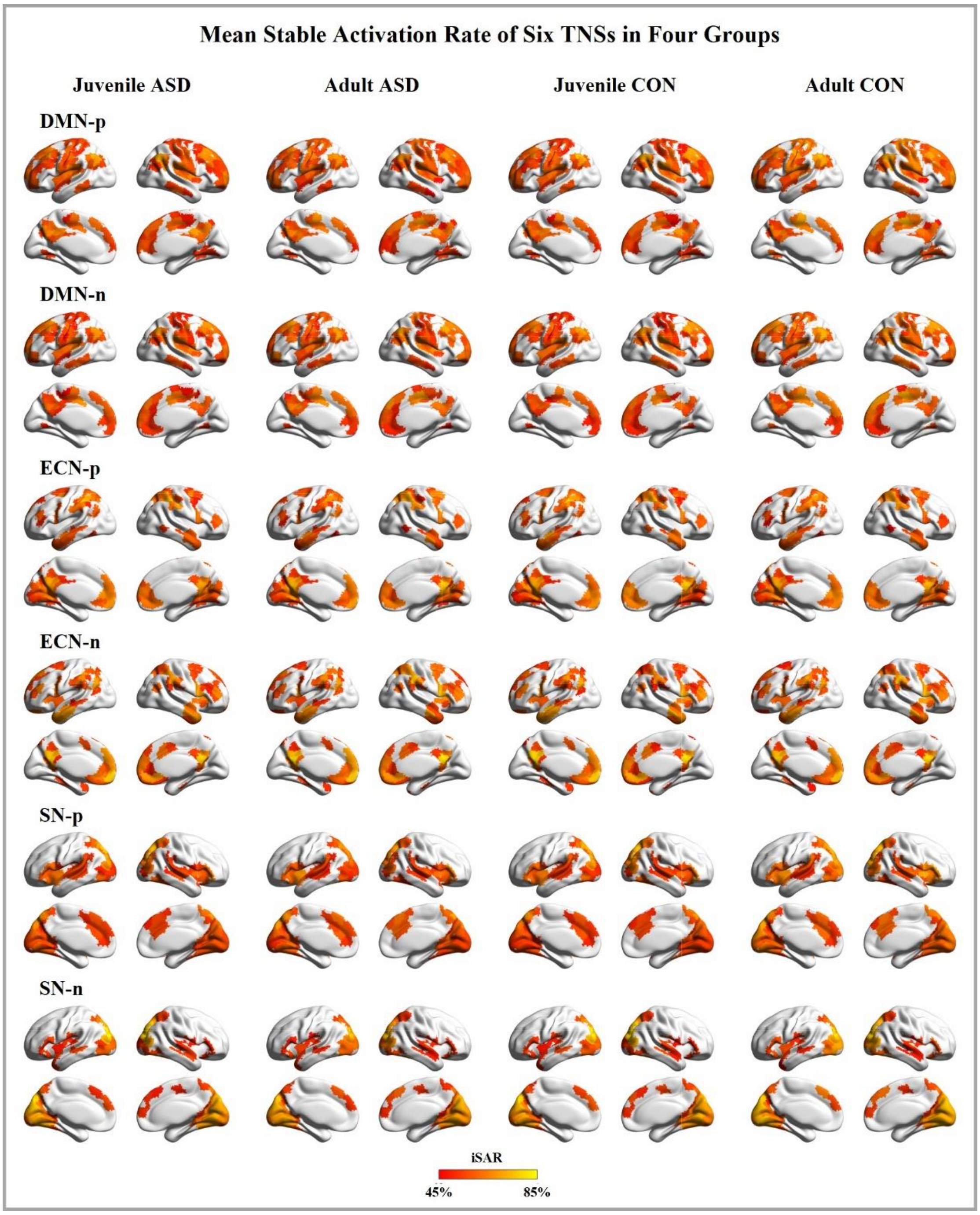

sFig. 8. Mean stable activation rate of six TNSs in four groups. For each TNS, every four graphs show the mean iSAR of stable regions for a group. From left to right, they are respectively juvenile ASD group, adult ASD group, juvenile CON group, and adult CON group.

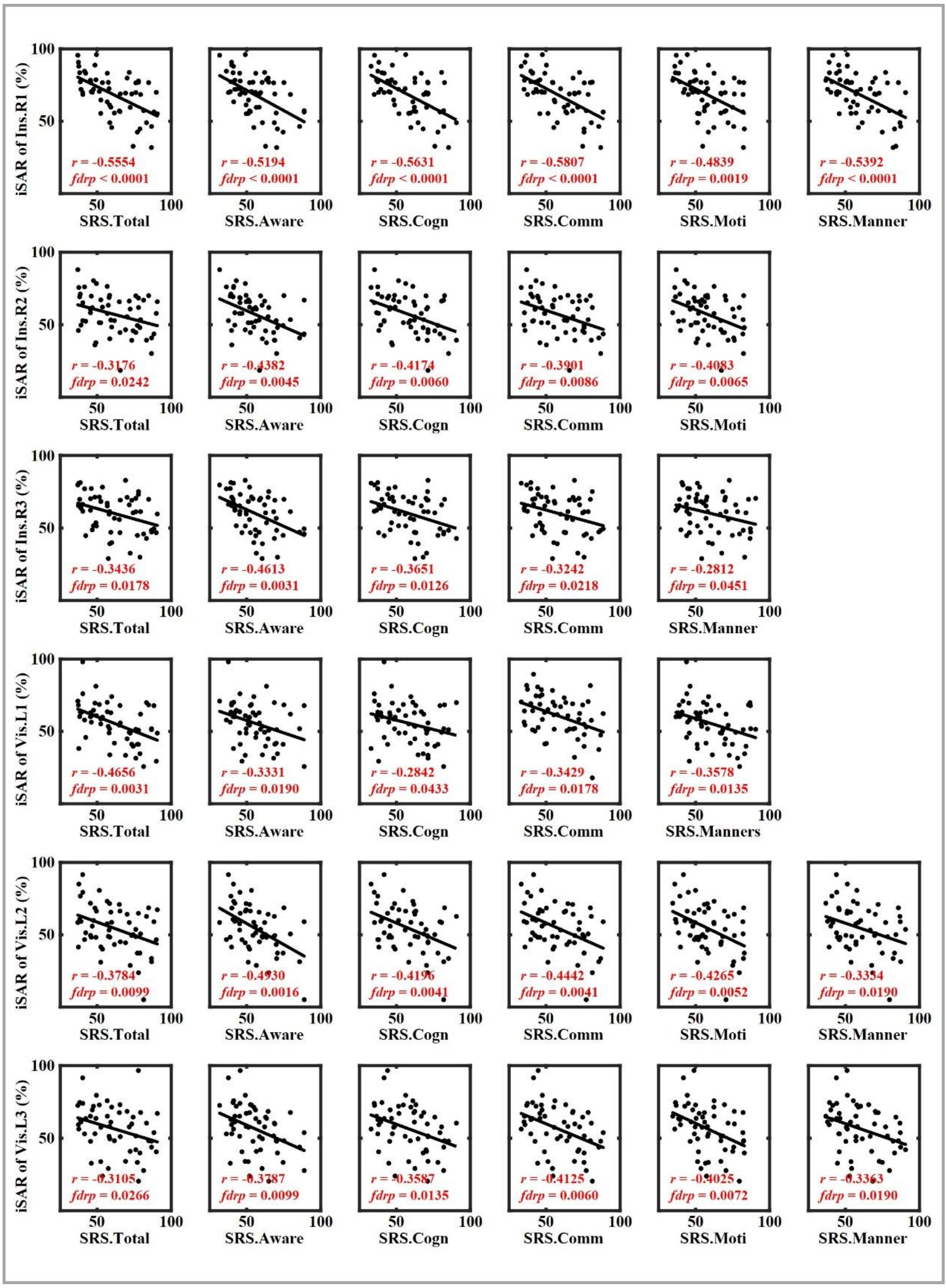

sFig. 9 Correlation between the SN-p iSAR values and SRS T scores, Part1. Results surviving FDR correction for Ins.L1-3 and Vis.L1-3 are displayed.

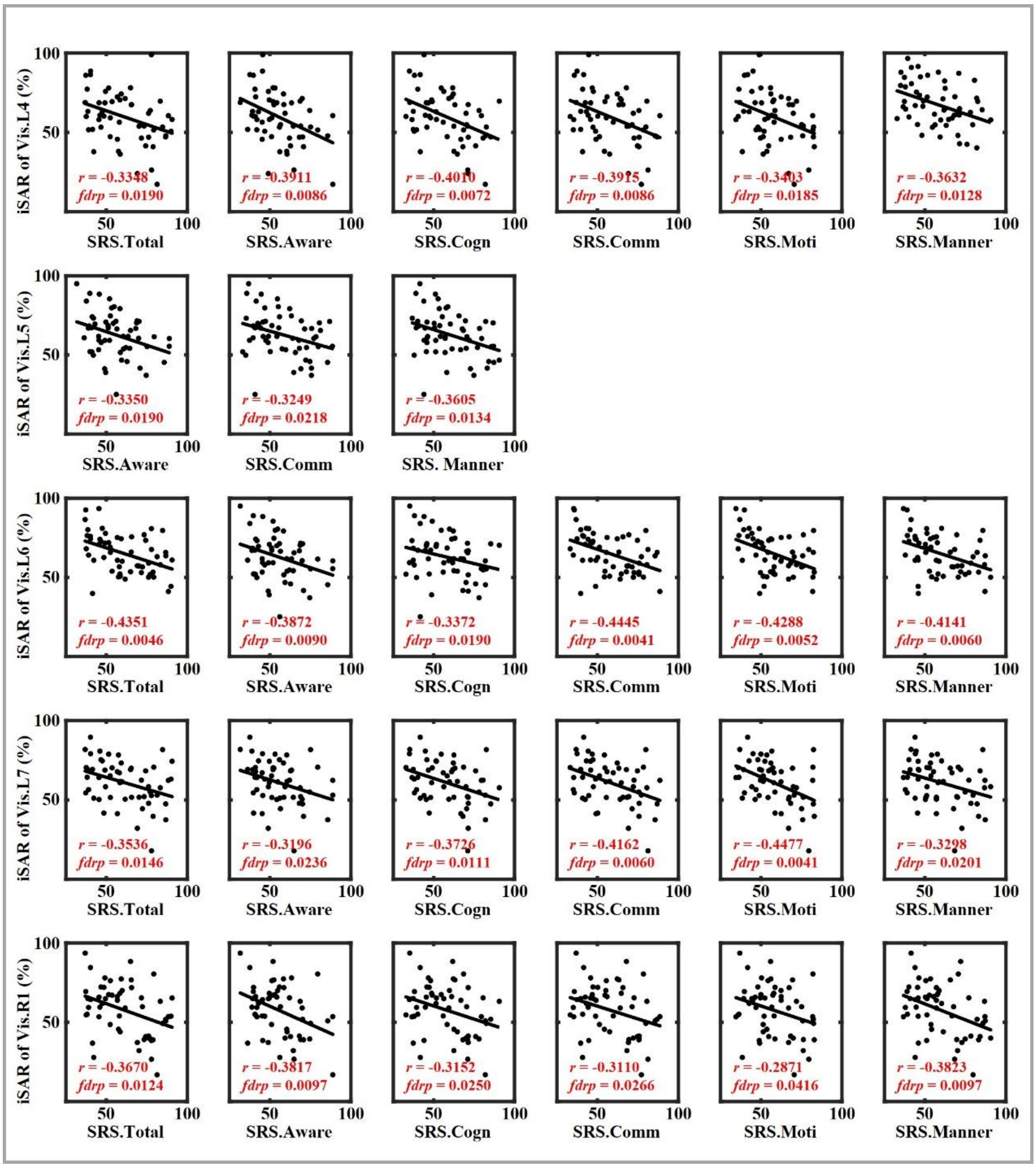

sFig. 10 Correlation between the SN-p iSAR values and SRS T scores, Part2.

Results surviving FDR correction for Vis.L4-7 and Vis.R1 are displayed.

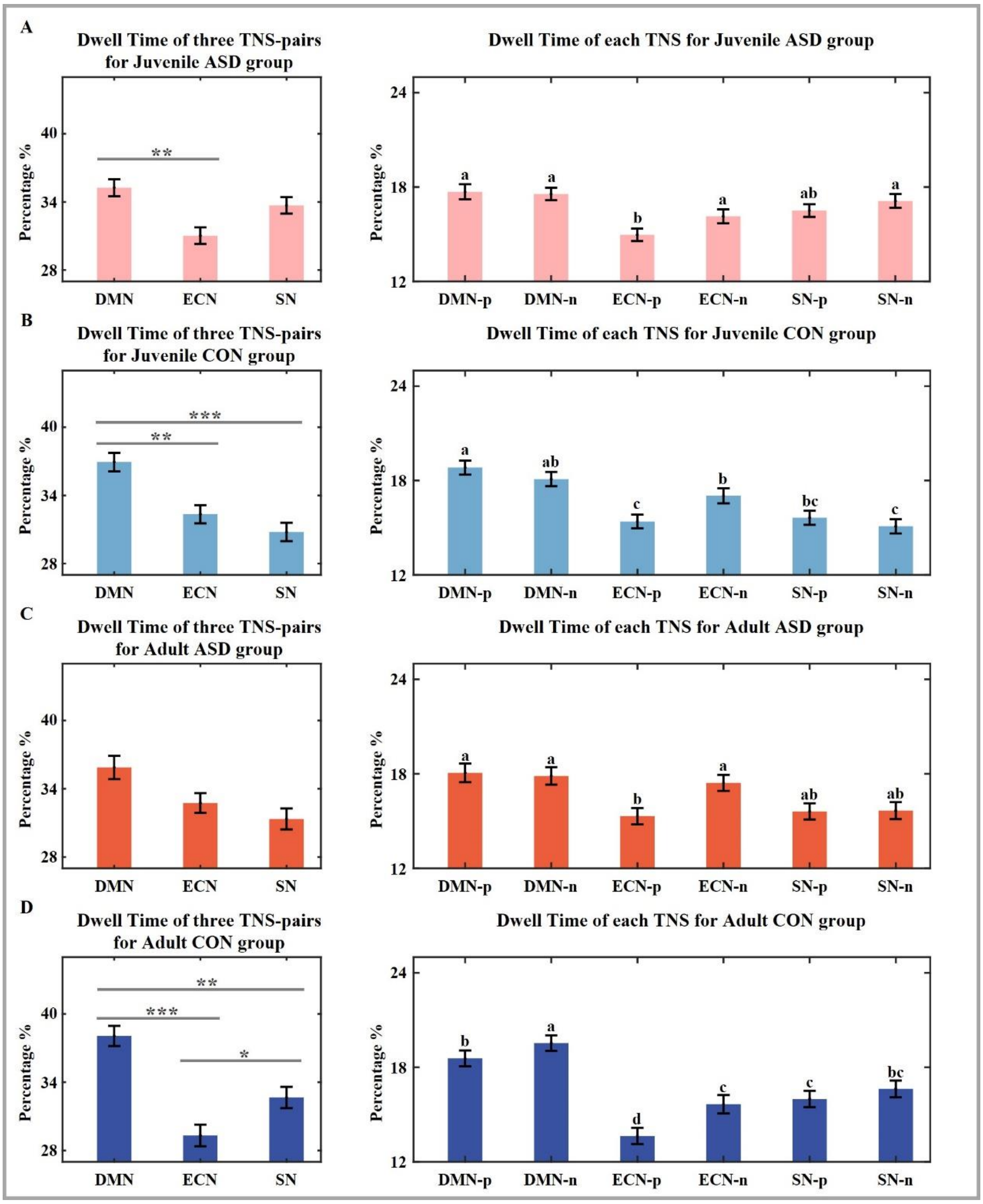

sFig. 11. Pairwise comparisons of TNS on dwell time for each group. The error-bar is standard error. TNSs have same labels means no significant difference (FDR corrected $p > 0.05$) between them. For example, in juvenile ASD group, 'ECN-p' and 'SN-p' both had label 'b', there is no difference between them.

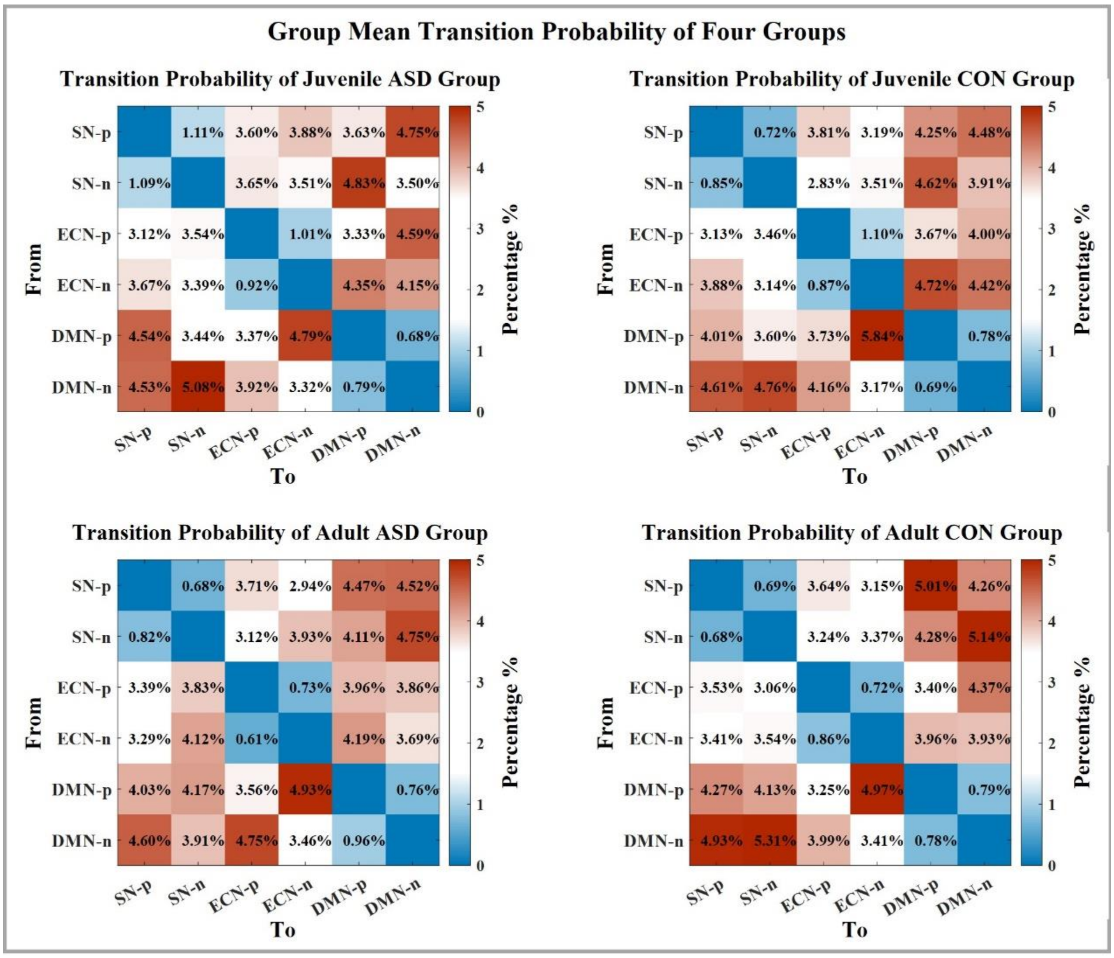

sFig. 12. Group mean transition probability of four groups.
